# Investigating the Role of MicroRNA and Transcription Factor Co-regulatory Networks in Multiple Sclerosis Pathogenesis

**DOI:** 10.3390/ijms19113652

**Published:** 2018-11-20

**Authors:** Nicoletta Nuzziello, Laura Vilardo, Paride Pelucchi, Arianna Consiglio, Sabino Liuni, Maria Trojano, Maria Liguori

**Affiliations:** 1 National Research Council, Institute of Biomedical Technologies, Bari Unit, 70126 Bari, Italy; nicoletta.nuzziello@gmail.com (N.N.); ariannaconsiglio@gmail.com (A.C.); sabino.liuni@ba.itb.cnr.it (S.L.); 2 National Research Council, Institute of Biomedical Technologies, Segrate Unit, 20090 Milan, Italy; laura.vilardo@itb.cnr.it (L.V.); paride.pelucchi@itb.cnr.it (P.P.); 3 Department of Basic Sciences, Neurosciences and Sense Organs, University of Bari, 70124 Bari, Italy; maria.trojano@uniba.it

**Keywords:** Multiple Sclerosis, miRNAs, transcription factors, target genes, bioinformatics, circulating biomarkers, pathogenesis

## Abstract

MicroRNAs (miRNAs) and transcription factors (TFs) play key roles in complex multifactorial diseases like multiple sclerosis (MS). Starting from the miRNomic profile previously associated with a cohort of pediatric MS (PedMS) patients, we applied a combined molecular and computational approach in order to verify published data in patients with adult-onset MS (AOMS). Six out of the 13 selected miRNAs (miR-320a, miR-125a-5p, miR-652-3p, miR-185-5p, miR-942-5p, miR-25-3p) were significantly upregulated in PedMS and AOMS patients, suggesting that they may be considered circulating biomarkers distinctive of the disease independently from age. A computational and unbiased miRNA-based screening of target genes not necessarily associated to MS was then performed in order to provide an extensive view of the genetic mechanisms underlying the disease. A comprehensive MS-specific miRNA-TF co-regulatory network was hypothesized; among others, SP1, RELA, NF-κB, TP53, AR, MYC, HDAC1, and STAT3 regulated the transcription of 61 targets. Interestingly, NF-κB and STAT3 cooperatively regulate the expression of immune response genes and control the cross-talk between inflammatory and immune cells. Further functional analysis will be performed on the identified critical hubs. Above all, in our view, this approach supports the need of multidisciplinary strategies for shedding light into the pathogenesis of MS.

## 1. Introduction

In humans, as in other species, the gene regulation process assumes multiple modes, including transcriptional regulation played by the regulatory proteins or transcription factors (TFs), and post-transcriptional regulation by including, most notably, microRNAs (miRNAs). MiRNAs are a class of highly conserved and single-stranded small non-coding RNAs that regulate gene expression by repressing specific target genes at the post-transcriptional level [1]. The miRNA repository miRBase (Release 22) [2] currently lists 1917 precursor miRNAs and 2654 mature miRNAs in the human genome; multiple or a cluster of miRNAs cooperatively regulate a given target gene leading to complex regulatory networks [3,4], and multiple genes can be targeted by the same miRNA. Taken together, these findings indicate that the relationships between miRNAs and their targets are not one-to-one, but multiple-to-multiple [5]. 

Besides miRNAs, TFs participate in the regulatory network that controls the expression of thousands mammalian genes by binding their promoter region at transcriptional level [6]. TFs and miRNAs are two key regulators that control their own expression and the expression of their mutual targets by feedback loop (FBL) and feed-forward loop (FFL) modalities [7]. FFLs can be distinguished into three types according to the master regulator: TF-FFL, miRNA-FFL, and composite FFL [8,9,10]. Recently, miRNA/TF-based FFLs have been reported to act as a major member of biological network motifs in complex multifactorial diseases like Multiple Sclerosis (MS) [11,12]. 

MS is a chronic inflammatory demyelinating and neurodegenerative disease of the Central Nervous System (CNS) [13] in which genetic, epigenetic, and environmental factors possibly contribute to its susceptibility and/or outcomes [14]. The disease onset typically occurs in young adults, especially females, although diagnosis during childhood and adolescence has been increasingly recognized worldwide, accounting for 3−10% of the whole MS population, hence called pediatric MS (PedMS) [15,16,17].

Given the complex heterogeneity of MS [18], the possibility to identify reliable markers, i.e., predictive of the clinical course or response to treatments, has so far been elusive due to its multifactorial nature that involves several genes and their interactions. To pursue this issue, in a previous study [19] we investigated the transcriptome profile of peripheral blood samples in a cohort of PedMS patients, and we found that 12 mature miRNAs were significantly upregulated (let-7a-5p, let-7b-5p, miR-25-3p, miR-125a-5p, miR-942-5p, miR-221-3p, miR-652-3p, miR-182-5p, miR-185-5p, miR-181a-5p, miR-320a, and miR-99b-5p) and one miRNA was downregulated (miR-148b-3p) in PedMS patients compared to pediatric control (PC) subjects. 

In the present evaluation, we analysed the expression pattern of the 13 identified miRNAs in an adult MS group from the same geographic area in order to verify whether the reported miRNomic profile was a peculiar signature of the “environmentally naïve” PedMS rather than a common feature of the general MS condition. 

Based on the obtained results and according to the transcriptional regulatory rule, a comprehensive MS-specific miRNA-TF co-regulatory network was then built, suggesting an alternative and valuable approach for the identification of significant regulators and their target genes in MS. 

## 2. Results

The study population consisted of 58 MS patients with adult onset of the disease (AOMS) and 20 age-matched healthy control (HC) subjects; Table 1 shows their demographic and clinical features. 

### 2.1. MicroRNA Differential Expression in AOMS

**Figure 1:**
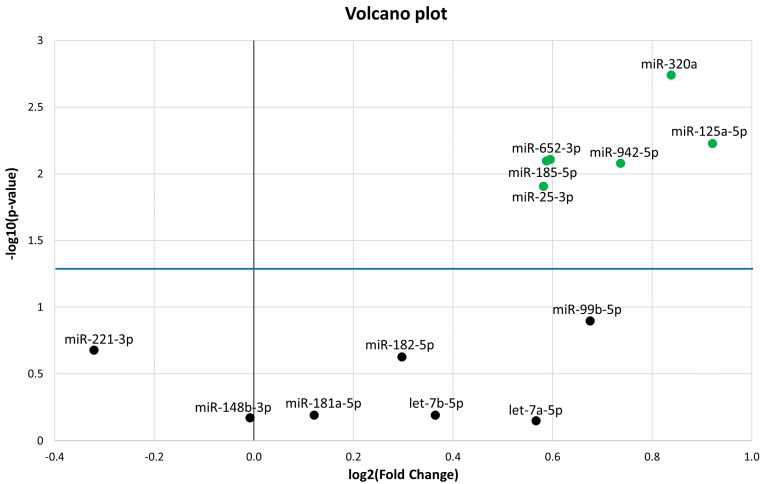
Volcano plot of the 13 microRNAs (miRNAs). The differentially expressed miRNAs with *p* < 0.05 are shown as green dots. All dots (black) under the blue line did not discriminate Multiple Sclerosis (MS) from healthy controls (HC). The Y-axis represents −log10 of the *p*-value and the X-axis represents log2 fold change of miRNA expression in the MS versus HC.

The 13 miRNAs that were differentially expressed (DE) in PedMS (details in Reference [19]) were analysed by a microfluidic qPCR approach in the AOMS patients. A normalization of each miRNA expression level was performed using the recommended reference miRNAs (miR-191-5p and miR-103a-3p), as suggested from the qPCR study [19,20,21], and from candidate miRNA endogenous controls in the TaqMan Advanced miRNA Assay white paper (Applied Biosystems, Thermo Fisher Scientific). Six miRNAs (miR-320a, miR-125a-5p, miR-652-3p, miR-185-5p, miR-942-5p, and miR-25-3p) were confirmed to be significantly DE in AOMS patients compared with HCs (Figure 1). In detail, results from the qPCR analysis showed statistically significant upregulation of miR-320a (FC = 1.79; *p*-value = 1.8 × 10^−3^), miR-125a-5p (Fold Change FC = 1.89; *p*-value = 5.9 × 10^−3^), miR-652-3p (FC = 1.51; *p*-value = 7.8 × 10^−3^), miR-185-5p (FC = 1.5; *p*-value = 8 × 10^−3^), miR-942-5p (FC = 1.67; *p*-value = 8.3 × 10^−3^) and miR-25-3p (FC =1.49; *p*-value = 1.2 × 10^−2^) in AOMS patients compared to HCs. 

**Figure 2:**
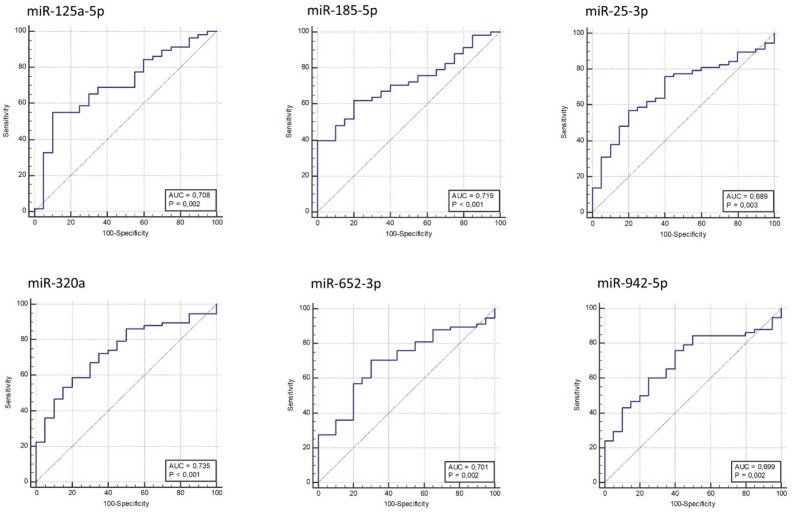
Receiver operating characteristic (ROC) curves for MS versus HC based on miRNAs relative expression data. The diagram is a plot of sensitivity (true-positive rate) versus specificity (false-positive rate).

A receiver operating characteristic (ROC) curve was generated for each validated miRNA, and the area under the curve (AUC) was calculated (Figure 2). MiR-320a, miR-185-5p, miR-125a-5p, and miR-652-3p provided AUC in a range from 0.701 to 0.735 (*p*-value ≤ 2 × 10^−3^), discriminating AOMS patients from HCs; miR-320a provided the best AUC (0.735; *p*-value = 1 × 10^−4^). 

### 2.2. MicroRNA Target Analysis

The identification of genes targeted by each miRNA is an important first step in elucidating its function(s). For this purpose, we determined the validated and predicted protein-coding gene targets of the six abovementioned DE miRNAs. Using databases containing experimentally validated miRNA-target interactions (miRtarbase and DIANA-Tarbase; see the Materials and Methods section), 155 miRNA-target pairs were validated by reporter gene assays. Since the prediction of the target site of existing algorithms can still be characterized by low precision and poor sensitivity, according to published guidelines [22], we integrated the predictions of different algorithms in order to combine their results. To this end, we uncovered 513 miRNA-target pairs predicted in at least four out of the five miRNA-target interaction tools (miRanda, RNA22, mirDB, TargetScan, DIANA-microT-CDS). Thirty miRNA-target pairs were found overlapping between the validated and predicted miRNA-target interactions. 

**Figure 3:**
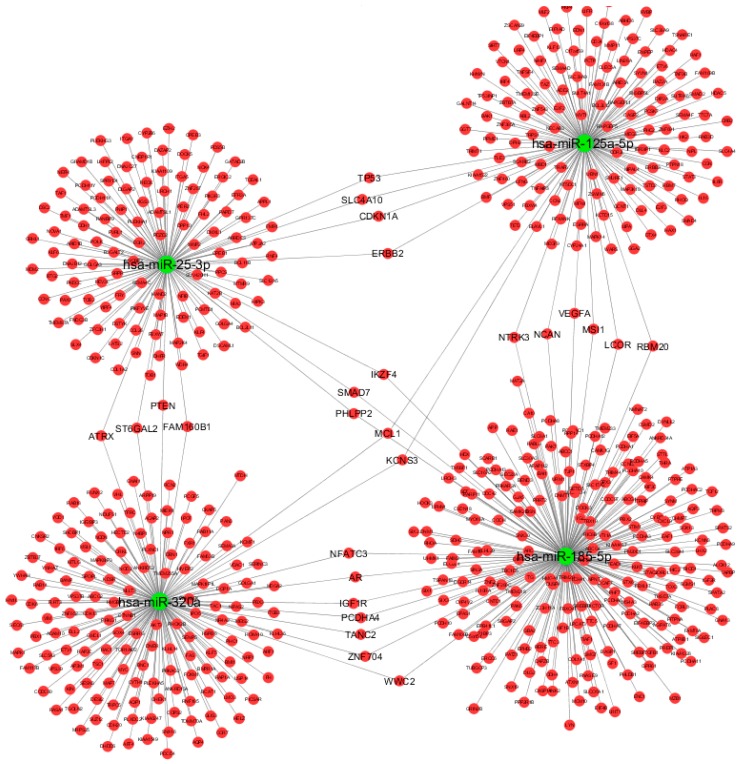
Graphical representation of computationally predicted/validated miRNA-target interactions using Cytoscape v3.6.0. Green nodes represent miRNAs, red nodes represent target genes. We excluded miR-942-5p and miR-652-3p miRNAs since our main purpose was to show the miRNA-based network.

We therefore constructed the miRNA-based network, including the DE miRNAs and their associated targets that fulfilled the selection criteria (Figure 3), using Cytoscape v3.6.0 [23]. The miRNA-target gene network consisted of 616 nodes (4 miRNAs and 612 miRNA targets) and 638 directed edges that resulted from miRNA-target interactions and were predicted by at least four algorithms and/or validated by reporter gene assays. Interestingly, several target genes (TP53, SLC4A10, CDKN1A, ERBB2, ATRX, ST6GAL2, PTEN, FAM160B1, SMAD7, IKZF4, PHLPP2, MCL1, KCNS3, NFATC3, AR, IGF1R, PCDHA4, TANC2, ZNF704, WWC2, NTRK3, NCAN, VEGFA, MSI1, LCOR, and RBM20) were shared by two of the following miRNAs: miR-125a-5p, miR-320a, miR-25-3p, and miR-185-5p. The remaining miR-942-5p and miR-652-3p were not enclosed in the network loop reported in Figure 3, as they did not share any target genes with the remaining miRNAs. 

### 2.3. miRNA-TF Co-regulatory Network 

**Figure 4:**
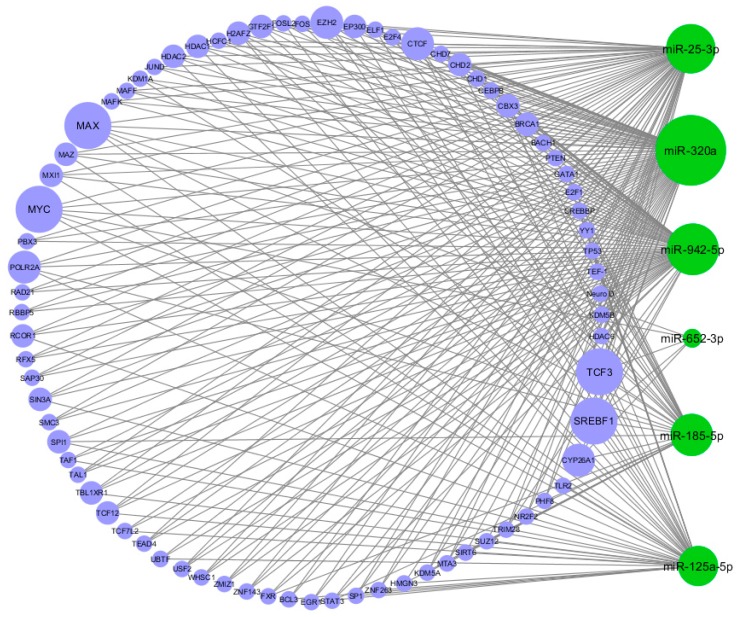
Circular view of transcription factor-miRNA (TF-miRNA) interactions. Green nodes represent the miRNAs, blue nodes represent the TFs. The size of the nodes is proportional to the degree of the nodes (number of incoming and outcoming edges). As shown in the figure, the four biggest TF nodes are MAX, MYC, TCF3, and SREBF1.

The TFs that regulate the six significantly DE miRNAs and their targets were identified. TF-miRNA interactions were exported from the Harmonizome [24] repository and combined with information on interactions from the TransmiR database [6]. We identified 409 TF-miRNA interactions (Figure 4); notably, MAX, MYC, TCF3, and SREBF1 were in common to all the six DE miRNAs. 

We also identified eight miRNA-TF feedback loops (FBLs) that included TP53, SREBF1, EZH2, FOXM1, MYC, ZBTB7A, SUZ12, miR-125a-5p, miR-185-5p, and miR-320a (Table 2). 

**Figure 5:**
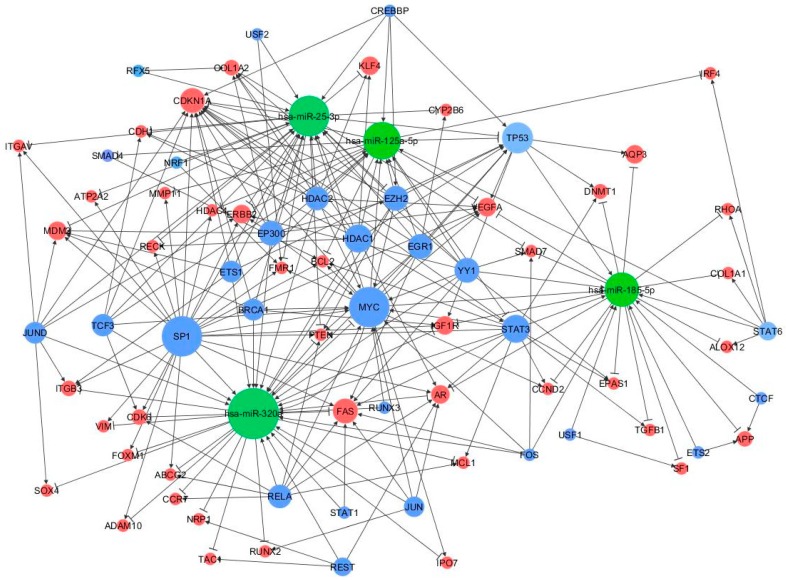
MiRNA-TF co-regulatory network. MiRNAs, genes and TFs are represented in green, red, and blue, respectively. Connections between the main players of the network are depicted by lines where the edges reflect the regulator mechanism, the arrows represent activation, and the perpendicular bars represent repression. The size of node is proportional to the degree of the node, i.e., the number of incoming and outgoing edges.

A peculiar miRNA-TF co-regulatory network was identified according to three-node feed-forward loops (FFLs): miRNA-FFL, TF-FFL, and composite FFL. The resultant network consisted of 93 nodes and 198 edges (Figure 5). 

Among the 198 FFLs, 21 were miRNA-FFLs (with miRNA acting as the main regulator), 152 belong to TF-FFLs (the main regulator here is the TF), and 25 were composite FFLs, where TF and miRNA regulate each other and their mutual targets. The nodes with the largest degree values (i.e., number of connections) were MYC, TP53, CDKN1A, SP1, and STAT3 (55, 47, 40, 36, and 34 edge connections, respectively), suggesting that they might be critical elements in the regulatory process of MS (FFL information are in Supplementary Table S1).

Among the above three subnetworks (miRNA-FFL, TF-FFL, and composite FFL), six genes (BCL2, MCL1, VEGFA, CDH1, CDK6, PTEN), three miRNAs (miR-125a-5p, miR-320a, miR-25-3p) and three TFs (MYC, STAT3, TP53) participated in all of them. 

### 2.4. Functional Enrichment and Pathway Analysis

Using the Database for Annotation, Visualization and Integrated Discovery (DAVID) [25] platform, we performed the functional enrichment analysis of all the nodes of the assembled miRNA-TF-based networks. The results showed several significant terms related to immunology, neurology, and inflammation processes (see Supplementary Figure S1 and Supplementary Table S2), e.g., immune system development (ad. *p*-value = 1.6 × 10^−11^), leukocyte differentiation (adj. *p*-value = 2.01 × 10^−6^), leukocyte activation (adj. *p*-value = 1 × 10^−3^), lymphocyte activation (adj. *p*-value = 3.5 × 10^−3^), lymphocyte differentiation (adj. *p*-value = 8.29 × 10^−6^), T cell differentiation (adj. *p*-value = 4.71 × 10^−4^), T cell activation (adj. *p*-value = 9.7 × 10^−3^), B cell differentiation (adj. *p*-value = 2.3 × 10^−3^), regulation of neurogenesis (adj. *p*-value = 1.07 × 10^−6^), neuron development (adj. *p*-value = 6.07 × 10^−4^), and regulation of cytokine production (adj. *p*-value = 1 × 10^−3^). 

An enrichment pathway analysis was also carried out among the identified hubs (Supplementary Table S3). The most significant were: the neurotrophin signalling pathway (adj. *p*-value = 6.08 × 10^−6^), the ErbB signalling pathway (adj. *p*-value = 6.19 × 10^−6^), the adherens junction (adj. *p*-value = 7.63 × 10^−5^), and the axon guidance (adj. *p*-value = 2.72 × 10^−4^) pathways.

### 2.5. Target Validation of miR-125a by Dual Luciferase Reporter Assay

The emerging importance of miR-125a-5p in the pathogenesis of MS led us to validate its predicted target genes related to mechanisms involved in MS. Therefore, in order to verify the ability of miR-125a-5p to post-transcriptionally downregulate putative targets through its binding to specific sites within their 3’ UTRs, a Dual Luciferase Reporter Assay was performed. The selected putative target genes assessed by this approach were DIP2A, ADD2, and E2F2.

**Figure 6:**
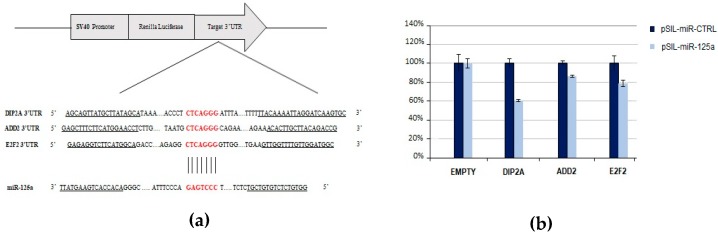
Experimental validation of miR-125a-5p and its predicted target genes by using luciferase reporter constructs. (**a**) Schematic representation of the psiCHECK2 luciferase reporter constructs containing the 3’UTR fragments of target genes cloned downstream the Renilla Luciferase gene. The sequence alignment between miR-125a seed region and target genes is reported. (**b**) The HEK-293T cells were co-transfected with four different experimental designs: (1) psiCHECK2-empty and pSIL-miR-CTRL; (2) psiCHECK2-empty and pSIL-miR-125a; (3) psiCHECK2-3’UTR and pSIL-miR-CTRL; and (4) psiCHECK2-3’UTR and pSIL-miR-125a. Y-axis represents Renilla Luciferase activity (%) normalized to Firefly Luciferase activity. Data represent the averages of at least three independent experiments with their standard deviations. The *p*-value was calculated by *t*-test. * *p*-value < 0.05.

We found that miR-125a caused a significant reduction of the normalized luciferase expression of DIP2A (39.6%, *p*-value = 6.8 × 10^−4^), ADD2 (13.4%, *p*-value = 3 × 10^−4^), and E2F2 (21.2%, *p*-value = 4.9 × 10^−3^) with respect to the control miRNA (Figure 6), thus indicating that the selected transcripts can be directly targeted by miR-125a.

## 3. Discussion

Six miRNAs (miR-320a, miR-125a-5p, miR-652-3p, miR-185-5p, miR-942-5p, and miR-25-3p) were significantly upregulated in both pediatric and adult MS patients, suggesting that they may be considered as circulating biomarkers distinctive of the disease, independently from the age of disease onset. Some of these miRNAs (miR-320a, miR-125a-5p, and miR-25-3p) have been already reported as dysregulated in published MS studies [9,25,26,27], the partial overlap possibly explained, e.g., by differences in the origin of the examined biological samples (blood cells [28,29,30,31], brain lesions [32,33], circulating extracellular vesicles [34,35]), or in the method of miRNAs quantification (qPCR, microarray, NGS). The remaining seven miRNAs previously associated with PedMS (let-7a-5p, let-7b-5p, miR-221-3p, miR-182-5p, miR-181a-5p, miR-99b-5p, miR-148b-3p) were not confirmed in AOMS, thus requiring a larger sample size to verify whether they were false positives, or more likely, they represent a molecular signature of the very early onset of the disease, in which a minimal environmental influence may enlighten the genetic load of MS [36]. Furthermore, it would be interesting to analyse the significantly DE miRNAs in the peripheral blood samples of patients with other inflammatory and neurodegenerative diseases in order to verify whether they are distinctive of MS rather than associated with common pathogenic pathways.

Among the DE miRNAs, miR-125a-5p was the most frequently associated with MS [9,37,38,39], and recently it has been implicated in the maturation of oligodendroglia [39]. Furthermore, the level of this miRNA was found abnormally high in cerebrospinal fluid (CSF), blood cells, and brain lesions of MS patients [40,41], suggesting that its pathological upregulation might contribute to the development of MS, leading, e.g., to an impaired repair of the demyelinated lesions. On these grounds, we tested (by luciferase reporter assay) the predicted interactions of miR-125a-5p with three predicted target genes (DIP2A, E2F2, ADD2), all related to mechanisms involved in MS and significantly downregulated in our previous study on PedMS subjects [19]. In our view, the implication of these three genes in the MS pathogenesis seems reasonable. DIP2A regulates both the synapse formation and the axonal path, and its expression was related to the burden of another neurodegenerative disorder, i.e. the Alzheimer’s disease [42]. The E2F2 gene was found to be involved in immunologic self-tolerance, the proliferation of autoreactive effector/memory T lymphocytes, and the B-cell differentiation [43,44]. Finally, ADD2 showed interactions with the alpha subunit of the Na^+^/K^+^-ATPase [45], whose dysfunction at the axonal level has been indicated as a major contributor to the progressive neurological decline observed in the chronic phases of MS [46]. Further studies are required to investigate the functional role of these validated miRNA/target regulations. Validations of the remaining genes targeted by the miRNAs of interest are ongoing in order to possibly identify other candidate genetic hubs in the pathogenic network of MS.

Another intriguing opportunity offered by this integrated computational approach was to identify the TF-miRNA co-regulatory networks in the peripheral blood samples of MS patients. SP1, RELA, NF-κB, TP53, AR, MYC, HDAC1, and STAT3 were the TFs that regulated 61 miRNA targets and were more frequently connected than other TFs. According to our data, a recently published study showed that the genes RELA, TP53, SP1, HDAC1, and AR seemed to be highly interactive, and demonstrated that a SP1-dependent transcription was able to modulate the autoimmune responses and played a central role in the MS pathogenesis [47]. Interestingly, NF-κB and STAT3 cooperatively regulate the expression of immune response genes and control the cross-talk between inflammatory and immune cells [48].

On the other hand, MAX, MYC, TCF3, and SREBF1 were TFs in common with the six DE miRNAs of this study. Since they were reported to be implicated in a miRNA-TF co-regulatory axis in prostate cancer [49], in our view it is reasonable to hypothesize that they may act together in the regulation of miRNAs transcription also in other pathological conditions like MS.

Finally, we identified the TF and miRNA mediated regulatory motifs, including FBLs and FFLs, which are promising regulators in MS. The FFL, a three-node motif pattern, is composed of two input elements (TF and miRNA) regulating each other and jointly impacting their target gene [50]. Although TFs and miRNAs seem to cooperate in the framework of a multigene transcriptional and post-transcriptional FFL, as far as we know, this regulatory mechanism has been poorly investigated in MS. In the MS network that was outlined in our analysis, many FFLs were TF-FFLs in which the TFs regulated either the miRNA or the gene; the resultant miRNA-TF co-regulatory network showed some critical hubs associated with MS. In particular, the nodes with the largest degree values were MYC, CDKN1A, TP53, SP1, and STAT3, suggesting that they may be crucial in the regulatory process of MS. Accordingly, Freiesleben et al. [11] identified 107 MS specific FFLs, which involved SP1, CDKN1A, TP53, and miR-125a-5p as critical nodes in the regulatory network motifs of the disease.

Among the other above-mentioned genes, BCL2 was reported as highly expressed in RRMS patients [51], while STAT3, MYC, JUN, NF-κB, and PTEN were identified as candidate genes for the MS susceptibility [52]. The study of Kristjansdottir et al. showed that an increased amount of transcription factor SP1 binds the risk allele of the CGGGG indel polymorphism significantly associated with MS [53], whereas TCF3 was found to be implicated in the gene transcription regulation observed in the pathogenic mechanisms of the disease [54]. Therefore, although we performed a computational miRNA-based screening of target genes not necessarily associated with MS, we found that the list of the critical hubs identified in this study was significantly enriched in MS-related genes. In our view, this represents a further support for the computational approach that we have applied, since it is independent from any pre-existing bias of other published MS studies.

Functional studies are needed to validate each segment of the identified hubs (miRNAs, TFs, and their targets); meanwhile, a pathway enrichment analysis was carried out among them in order to possibly identify common affected pathways already associated with MS. The most significant was the neurotrophin signalling pathway that recruited four DE miRNAs (miR-125a-5p, miR-320a, miR-185-5p, and miR-25-3p), 18 miRNA targets, and six TFs (RELA, TP53, NF-κB1, FOXO3, JUN, ABL1), thus confirming the pivotal role of neurotrophin in neuroprotective and neurodegeneration processes of MS [55]. Furthermore, they induce the release of neuroregulins, mediators of their biological activity through the binding of the receptor tyrosine kinases that encloses the ErbB genes [56,57]. The ErbB receptor family is an important regulator of oligodendrogenesis, myelination, and serotonin brain levels [58]; interestingly, we found that the ErbB signalling was the second most significant canonical pathway resulting from our data. On the other hand, a Gene Ontology (GO) term enrichment analysis showed that all the DE miRNAs, TFs, and their target genes were involved in important immune functions and in the maintenance of neuronal homeostasis. For example, miR-125a-5p and its validated target gene ADD2 were found to be implicated in the immune system development; furthermore, miR-125a-5p targeted multiple genes involved in both immunology-related (e.g., T and B cell differentiation) and neurology-related terms (e.g., neuron differentiation and development).

In conclusion, in this study we were able to draw a systematic, comprehensive, and complex predictive network associated with MS, in which some hubs, represented by miRNAs and FFLs, seem to play important roles in the regulatory networks underlying the disease pathogenesis. Since the significant TFs and target genes were not preliminarily selected as associated with MS, we used very stringent bioinformatic criteria to detect the relevant hubs while avoiding false positive outcomes. We applied a computational hypothesis-free, unbiased approach that overcomes the gaps imposed by the incomplete understanding of this complex multifactorial disease. In fact, we believe that restricting the investigation to a pre-selection of only known MS-associated genetic elements might influence the results and provide only a partial view of the disease mechanisms that still remain largely unknown. Further functional analyses are ongoing in order to investigate and clarify the role of the identified hubs in MS pathogenesis.

Above all, our study identified candidate gene regulatory network motifs, which might improve the understanding of gene regulation mechanisms in MS; additionally, a comparison between the molecular profile (miRNA expression) of pediatric and adult MS was performed, revealing some discrepancies that, if confirmed in larger populations, may be the resulting effect, e.g., of environmental factors in the epigenetic components of this complex disease. 

## 4. Materials and Methods

### 4.1. Study Population

Caucasian patients with adult onset MS [59,60] were recruited at the Department of Basic Sciences, Neurosciences and Sense Organs, University of Bari (Italy).

Age-matched healthy controls (HCs) were recruited among volunteers who did not show clinical signs or instrumental evidences of inflammatory or neurological diseases, and who had negative family histories for MS and other neurodegenerative diseases.

All experiments were performed according to relevant guidelines/regulations and approved by the local ethical committee of the Azienda Ospedaliera Policlinico Universitario of Bari (Italy) (prot. 0070059/CE, 18-09-2015). Written informed consent was obtained from all participants (under the Declaration of Helsinki statement).

### 4.2. Sample Preparation

Peripheral blood samples were collected in PAXgene Blood RNA Tubes (PreAnalytiX Qiagen/BD, Hombrechtikon, Switzerland), coded, anonymized, and frozen at −20 °C.

Total RNA was isolated by using PAXgene Blood RNA Kit (PreAnalytiX Qiagen/BD, Hilden, Germany). RNA purification was performed using the manual procedure, according to the manufacturer’s protocol. RNA quantity and quality were assessed by Nanodrop ND-1000 (Thermo Fisher Scientific, Wilmington, DE, USA) and RNA 6000 Pico chip on Bioanalyzer 2100 (Agilent Technologies, Santa Clara, CA, USA), respectively.

### 4.3. Reverse Transcription and Microfluidic qPCR

TaqMan Advanced miRNA Cards (Applied Biosystems, Thermo Fisher Scientific) were employed for the quantitative analysis of the 13 miRNA expressions. The input RNA was reverse transcribed using a TaqMan Advanced miRNA cDNA synthesis kit (Applied Biosystems, Thermo Fisher Scientific), and then subjected to qPCR in triplicate using the TaqMan microfluidic cards, according to the manufacturer’s protocol. The TaqMan card is a custom designed miRNA assay, preconfigured in a 384-well format and spotted onto a microfluidic card. The card enables the simultaneous quantitation of 13 human miRNAs plus 2 endogenous reference miRNAs for 8 samples per card (3 replicates per assay). Briefly, 25 µL of diluted pre-amplified product was mixed with 50 µL of TaqManVR Fast Advanced Master Mix (Applied Biosystems, Thermo Fisher Scientific) and 25 µL of nuclease-free water; 100 µL of the PCR reaction mix was dispensed into each port of the TaqMan Advanced miRNA Card. Each card was centrifuged, sealed with a TaqMan low-density array sealer (Applied Biosystems, Life Technologies), and placed in the microfluidic card sample block of an ABI PrismVR 7900HT sequence detection system (Applied Biosystems, Life Technologies). Finally, PCR amplification was performed. Thermal cycling parameters were 10 min at 92 °C to enzyme activation, 40 cycles of denaturation at 95 °C for 1sec, and annealing and extension at 60 °C for 20 s.

Raw Ct values were calculated using RQ manager software v.2.3 (ABI).

### 4.4. Statistical Analysis

The relative expression levels of each miRNA, normalized to the geometric mean of endogenous reference miRNAs (miR-191-5p and miR-103a-3p), were calculated using the formula 2^−ΔΔCT^, as in equation (1), where

ΔΔCt = (Ct_Target_ − Ct_References_)_MS_ − (Ct_Target_ − Ct_References_)_Calibrator_ (1)

Each sample was replicated three times. The cycle number at which the reaction crossed an arbitrarily placed threshold (Ct) was determined for each miRNA. We used Ct = 40 as a cut-off. The Mann–Whitney test was performed to identify differentially expressed miRNAs between MS patients and HC subjects. *p*-value < 0.05 was considered statistically significant.

The receiver operating characteristic curve (ROC) is a graphical approach for investigating sensitivity and specificity, and the area under ROC (AUC) provides an estimate of the miRNA’s ability to discriminate the compared groups. ROC curve analysis was performed, and AUC was calculated using MedCalc Software. The associated *p*-values and AUCs were calculated for each miRNA.

### 4.5. MicroRNA Target Analysis

Seven databases comprising predicted and validated miRNA-target interactions were used. miRanda (http://www.micro rna.org/microrna/home.do, latest access: 08-01-2018), DIANA-microT-CDS [61], rna22 (https://cm.jefferson.edu/rna22, latest access: 08-01-2018), mirDB (http://ophid.utoronto.ca/mirDIP, latest access: 08-01-2018), and TargetScan (http://www.targetscan.org/vert_71, latest access: 11-01-2018) collect predicted miRNA targets. Two databases, miRtarbase (http://mirtarbase.mbc.nctu.edu.tw, latest access: 14-01-2018) and DIANATarbase (http://diana.imis.athena-innovation.gr/DianaTools/index.php?r¼site/index, latest access: 14-01-2018), contain validated miRNA targets.

In order to reduce the probability of false positives and/or negatives, only those bindings that were confirmed by reporter gene assays in the previously mentioned databases or computationally predicted at least by 4 algorithms were finally selected.

### 4.6. Transcription Factor Target Analysis

TF-miRNA interactions were exported from the CHEA [62], ENCODE [63], TRANSFAC [64], MotifMap [65], and JASPAR [66] datasets obtained from the Harmonizome [24] repository, and combined with information on interactions from TransmiR database [6].

TF-mRNA interactions were exported from the TRRUST v.2 database (www.grnpedia.org/trrust), which makes use of a text-mining algorithm coupled with manual curation of the results to populate a database of 8444 regulatory interactions [67].

Finally, miRNA-TF co-regulatory networks were constructed according to the miRNA-based FFL, TF-based FFL, and miRNA-TF FBLs. These networks were visualized using Cytoscape v3.6.0 [23].

### 4.7. Functional and Pathway Analysis

To identify genes functionally related to miRNA- or TF-based networks, we performed a functional analysis (GO-term) using the Database for Annotation, Visualization and Integrated Discovery (DAVID v6.8, http://david.abcc.ncifcrf.gov, latest access: 16-01-2018) tool. We adjusted the *p*-value of the enriched terms for multiple testing using the Benjamini-Hochberg correction (adjusted *p*-value < 0.05). We also performed a pathway analysis using the DAVID tool and adjusted the *p*-value of the enriched pathway using the Benjamini-Hochberg correction (adjusted *p*-value < 0.05). 

### 4.8. Luciferase Reporter Assay

The human HEK-293T cell line, purchased from the American Type Culture Collection (ATCC, MD) was cultured in DMEM/High Glucose medium (Thermo Fisher Scientific) supplemented with 10% fetal bovine serum (FBS, Thermo Fisher Scientific) and 100 U/mL penicillin/streptomycin (Thermo Fisher Scientific) in 5% CO2 at 37 °C.

To construct luciferase reporter vectors, the 3′UTR fragments of target genes containing the miR-125a putative binding site were amplified by PCR using Platinum Taq DNA Polymerase (Thermo Fisher Scientific) and cloned into psiCHECK-2 vector (Promega, Madison, Wisconsin, USA) downstream the Renilla luciferase reporter gene. Primers (5′ to 3′) used for cloning DIP2a were: For GGTCTCGAGCAGTTATGCTTATAGCA and Rev CCAGCGGCCGCACTTGATCCTAATTTTGTAA; primers (5′ to 3′) used for cloning ADD2 were: For GCTCTCGAGCTTTCTTCATGGAACCT and Rev CGTGCGGCCGCGGTCTGTAAGCAAGTGT; primers (5′ to 3′) used for cloning E2F2 were: For GGACTCGAGAGGTCTTCATGGCA and Rev GGAGCGGCCGCCATCCAACAAAACCAAC. The vectors were obtained cloning PCR products into psiCHECK-2 plasmid using XhoI and NotI restriction enzymes.

To construct the miR-125a expression plasmid, the pre-miR-125a and its flanking regions were obtained by PCR amplification using Platinum Taq DNA Polymerase (Thermo Fisher Scientific) and cloned into pSilencer (pSIL) 4.1-CMV neo vector (Thermo Fisher Scientific). Primers (5′ to 3′) used for cloning miR-125a were: For TCTCTGCTGTGTCTCTGTGG and Rev TTATGAAGTCACCACAGGGC. The vectors were obtained by cloning PCR products into psiCHECK-2 plasmid using BamHI and HindIII restriction enzymes. Plasmid construct sequences were verified by Sanger sequencing.

Lipofectamine 2000 (Invitrogen, Thermo Fisher Scientific) was used for transfection following manufacturer’s instructions. Briefly, ~10,000 cells per well in 24-well plates were transfected with a mixture including 100 ng psiCHECK2-3′UTR construct and 300 ng pSIL-miR125a construct; for controls, 100 ng of psiCHECK2-empty (control vector without any cloned region) construct and 300 ng pSIL-miR-CTRL were used. Each transfection was repeated in triplicate. The dual-luciferase assays (Promega) were performed at 48 h after transfection and luminescence was measured using a Victor Light 1420 luminometer (Perkin Elmer).

The ratio (R/FF) of Renilla luciferase activity to Firefly luciferase activity (used as an internal control of the transfection efficiency) in each well was normalized to the average ratio of the control wells in each plate in which the ratio was designated as 1. All experimental data are presented as the mean ± SD from three independent transfection experiments. Statistical analysis was performed by Two-tailed Student’s *t*-tests and a *p*-value < 0.05 was considered statistically significant.

## Figures and Tables

**Table 1:** Summary of the demographic and clinical features of the study population.

	AOMS (no. 58)	HC (no. 20)
**Female/Male Ratio**	2.7	2.3
**Age (y, mean ± SD)**	37.8 ± 11.3	43.2 ± 3.1
**Age at MS Onset (y, mean ± SD)**	34.3 ± 9.6	
**MS Course (RR/SP/PP)**	54/4/0	
**Disease Duration (y, mean ± SD)**	13.4 ± 9.3	
**EDSS Score**	2.7 ± 1.1	
**DMT (y/n)**	56/2	

AOMS = Adult-Onset Multiple Sclerosis; HC = healthy control; DMT = disease modifying treatment.

**Table 2:** List of significantly upregulated miRNAs in the comparison between MS and HC. For each miRNA, the log2 fold change and *p*-value from qPCR analysis have been detailed. The ROC section shows the results of AUC and associated *p*-value. The total number of miRNA targets and TFs that regulate each miRNA has been indicated. In the last section (miRNA-TF co-regulatory networks), we listed the total number of networks in which the studied miRNAs seem to be involved.

miRNA	qPCR		ROC		Target *	TF	miRNA-TF co-regulatory Networks			
	logFC	*p*-value	AUC	*p*-value			FBL	miRNA-FFL	TF-FFL	Composite FFL
miR-320a	0.8383653	0.0018	0.735	0.0001	157	141	4	6	52	9
miR-125a-5p	0.9214846	0.0059	0.708	0.0016	140	40	2	9	28	6
miR-652-3p	0.5953385	0.0078	0.701	0.0017	2	5				
miR-185-5p	0.5878233	0.0080	0.719	0.0002	220	49	1	2	20	
miR-942-5p	0.7367637	0.0083	0.699	0.0016		95				
miR-25-3p	0.5819802	0.0124	0.689	0.0034	119	79	1	4	52	10

Abbreviations: ROC = receiver operating characteristic; AUC = area under the receiver operating characteristic; TF = transcription factor; FBL = feedback loop; FFL = feed-forward loop. * Experimentally validated by reporter gene assays or computationally predicted at least by four algorithms.

## Supplementary Materials

Supplementary materials can be found at http://www.mdpi.com/1422-0067/19/11/3652/s1.

## Abbreviations

miRNA	microRNA
TF	Transcription Factor
MS	Multiple Sclerosis
PedMS	Pediatric Multiple Sclerosis
AOMS	Adult-Onset Multiple Sclerosis
FBL	Feedback Loop
FFL	Feed-Forward Loop
CNS	Central Nervous System
HC	Healthy Control
DE	Differentially Expressed
ROC	Receiver Operating Characteristic
AUC	Area Under the Curve
R	Renilla
FF	Firefly
