# Supplementary material for: Investigating the Role of MicroRNA and Transcription Factor Co-regulatory Networks in Multiple Sclerosis Pathogenesis
Source: Int J Mol Sci. 2018 Nov 20;19(11):3652. doi: 10.3390/ijms19113652 (PMC6274935; doi:10.3390/ijms19113652)
Supplement: Supplementary file 1 [file ijms-19-03652-s001.pdf]

Supplementary Material

Investigating the role of microRNA and Transcription Factor co-regulatory networks in Multiple Sclerosis pathogenesis

Nicoletta Nuzziello[1], Laura Vilardo[2], Paride Pelucchi[2], Arianna Consiglio[1], Sabino Liuni[1], Maria Trojano[3] and Maria Liguori[1]*

[1]National Research Council, Institute of Biomedical Technologies, Bari Unit, Bari, Italy
[2]National Research Council, Institute of Biomedical Technologies, Segrate Unit, Milan, Italy
[3]Department of Basic Sciences, Neurosciences and Sense Organs, University of Bari, Bari, Italy

Supplementary Figure S1

Frequencies of GO terms and canonical pathways. **(a)** Histogram illustrates the GO terms associated to assembled sub-networks. **(b)** Histogram illustrates the canonical pathways associated to assembled sub-network.

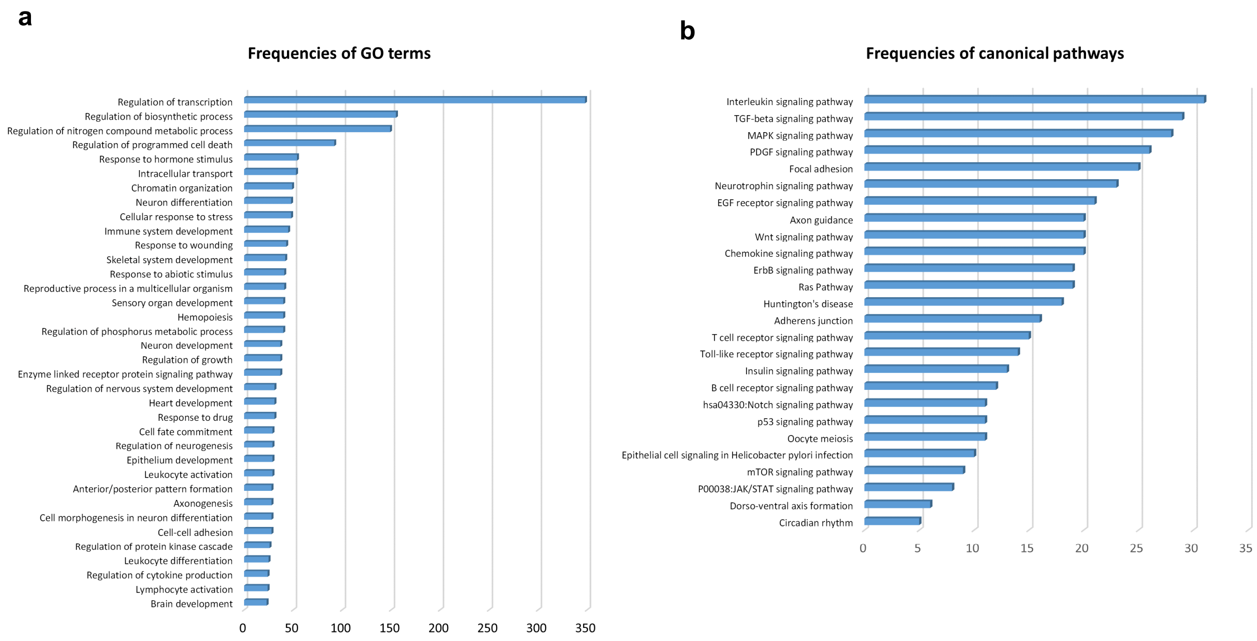

Legends for Supplementary Tables

Supplementary Table S1

List of feedback (FBL) and feed-forward (FFL) loops in miRNA-TF co-regulatory network.

Supplementary Table S2

List of significantly (adj p-value < 0.05) GO-term involved in MS. The first column (from the left) listed the GO-term (biological processes) involved in MS. For each functional class, the main attributes (gene count, p-value, adjusted p-value of the enriched terms for multiple testing using the Benjamini correction) have been detailed. In the last column (on the right), we summarized the target genes involved in each enriched GO-term.

Supplementary Table S3

List of significantly (adj p-value < 0.05) enriched pathway involved in MS. The first column (from the left) listed the enriched pathway involved in MS. For each pathway, the main attributes (gene count, p-value, adjusted p-value of the enriched terms for multiple testing using the Benjamini correction) have been detailed. In the last column (on the right), we summarized the target genes involved in each enriched pathway.

Supplementary Table S1

TF-FFL			miRNA-FFL			composite FFL			FBL	
**FT**	**miRNA**	**Target**	**miRNA**	**target**	**TF**	**miRNA**	**target**	**TF**	**miRNA**	**TF**
CREBBP	miR-125a-5p	CDKN1A	miR-125a-5p	BCL2	STAT3	miR-125a-5p	BCL2	TP53	miR-125a-5p	TP53
CREBBP	miR-125a-5p	TP53	miR-125a-5p	BCL2	SMAD4	miR-125a-5p	CDKN1A	TP53	miR-125a-5p	ZBTB7A
EGR1	miR-125a-5p	CDKN1A	miR-125a-5p	CDKN1A	TP53	miR-125a-5p	E2F3	TP53	miR-185-5p	SREBF1
EGR1	miR-125a-5p	TP53	miR-125a-5p	CDKN1A	STAT3	miR-125a-5p	MCL1	TP53	miR-25-3p	EZH2
EGR1	miR-125a-5p	VEGFA	miR-125a-5p	CDKN1A	HDAC4	miR-125a-5p	STAT3	TP53	miR-320a	FOXM1
EP300	miR-125a-5p	CDKN1A	miR-125a-5p	CDKN1A	SMAD4	miR-125a-5p	VEGFA	TP53	miR-320a	MYC
EP300	miR-125a-5p	ERBB2	miR-125a-5p	MCL1	STAT3	miR-25-3p	CDH1	EZH2	miR-320a	PBX3
EP300	miR-125a-5p	VEGFA	miR-125a-5p	SMAD4	HDAC4	miR-25-3p	CDKN1A	TP53	miR-320a	SUZ12
EZH2	miR-125a-5p	CDKN1A	miR-125a-5p	VEGFA	HDAC4	miR-25-3p	EZH2	TP53		
EZH2	miR-125a-5p	TP53	miR-185-5p	STIM1	AR	miR-25-3p	MDM2	TP53		
HDAC1	miR-125a-5p	CDKN1A	miR-185-5p	VEGFA	AR	miR-25-3p	PTEN	TP53		
HDAC1	miR-125a-5p	STAT3	miR-25-3p	CDH1	KLF4	miR-25-3p	TCEAL1	TP53		
HDAC1	miR-125a-5p	TP53	miR-25-3p	CDKN1A	KAT2B	miR-25-3p	CDKN1A	EZH2		
HDAC2	miR-125a-5p	VEGFA	miR-25-3p	CDKN1A	KLF4	miR-25-3p	CPEB1	EZH2		
SP1	miR-125a-5p	BCL2	miR-25-3p	CDKN1C	KLF4	miR-25-3p	PTEN	EZH2		
SP1	miR-125a-5p	CDKN1A	miR-320a	CDK6	AR	miR-320a	AR	FOXM1		
SP1	miR-125a-5p	ERBB2	miR-320a	CTNNB1	AR	miR-320a	MYC	FOXM1		
SP1	miR-125a-5p	HDAC4	miR-320a	IGF1R	AR	miR-320a	BMI1	MYC		
SP1	miR-125a-5p	MMP11	miR-320a	MYC	AR	miR-320a	CDK6	MYC		
SP1	miR-125a-5p	VEGFA	miR-320a	PTEN	AR	miR-320a	FOXM1	MYC		
SPI1	miR-125a-5p	SCARB2	miR-320a	VIM	AR	miR-320a	MAPK1	MYC		
YY1	miR-125a-5p	ERBB2				miR-320a	MTDH	MYC		
MYC	miR-125a-5p	BCL2				miR-320a	TFRC	MYC		
MYC	miR-125a-5p	CDKN1A				miR-320a	CHEK1	MYC		
MYC	miR-125a-5p	TP53				miR-25-3p	BTG2	TP53		
MYC	miR-125a-5p	VEGFA								
TCF3	miR-125a-5p	CDKN1A								
STAT6	miR-125a-5p	IRF4								
BRCA1	miR-320a	IGF1R								
BRCA1	miR-320a	MYC								
EGR1	miR-320a	FAS								
EGR1	miR-320a	PTEN								
EP300	miR-320a	MYC								
ETS1	miR-320a	ITGB3								
EZH2	miR-320a	MYC								
EZH2	miR-320a	PTEN								
FOS	miR-320a	FAS								
FOS	miR-320a	MYC								
HDAC1	miR-320a	AR								
HDAC2	miR-320a	MYC								
JUN	miR-320a	AR								
JUN	miR-320a	FAS								
JUN	miR-320a	MYC								
JUN	miR-320a	RUNX2								
JUND	miR-320a	ITGB3								
RELA	miR-320a	ABCG2								
RELA	miR-320a	AR								
RELA	miR-320a	CDK6								
RELA	miR-320a	FAS								
RELA	miR-320a	MCL1								

RELA	miR-320a	MYC			
RELA	miR-320a	PTEN			
REST	miR-320a	AR			
REST	miR-320a	NRP1			
REST	miR-320a	TAC1			
RUNX3	miR-320a	MYC			
SP1	miR-320a	ABCG2			
SP1	miR-320a	AR			
SP1	miR-320a	CDK6			
SP1	miR-320a	FAS			
SP1	miR-320a	FOXM1			
SP1	miR-320a	IGF1R			
SP1	miR-320a	ITGB3			
SP1	miR-320a	MYC			
SP1	miR-320a	PTEN			
SP1	miR-320a	VIM			
STAT1	miR-320a	FAS			
STAT3	miR-320a	AR			
STAT3	miR-320a	FAS			
STAT3	miR-320a	MCL1			
STAT3	miR-320a	MYC			
STAT3	miR-320a	PTEN			
TCF3	miR-320a	MYC			
TCF3	miR-320a	CDK6			
YY1	miR-320a	FAS			
YY1	miR-320a	MYC			
JUND	miR-320a	SOX4			
MYC	miR-320a	IPO7			
RELA	miR-320a	CCR7			
SP1	miR-320a	ADAM10			
BRCA1	miR-25-3p	CDKN1A			
BRCA1	miR-25-3p	MDM2			
CREBBP	miR-25-3p	CDKN1A			
CREBBP	miR-25-3p	EZH2			
CREBBP	miR-25-3p	TP53			
EGR1	miR-25-3p	CDKN1A			
EGR1	miR-25-3p	CYP2B6			
EGR1	miR-25-3p	PTEN			
EGR1	miR-25-3p	TP53			
EP300	miR-25-3p	CDKN1A			
EP300	miR-25-3p	ERBB2			
EP300	miR-25-3p	EZH2			
EP300	miR-25-3p	MDM2			
ETS1	miR-25-3p	CDKN1A			
ETS1	miR-25-3p	MDM2			
ETS1	miR-25-3p	TP53			
HDAC1	miR-25-3p	CDH1			
HDAC1	miR-25-3p	CDKN1A			
HDAC1	miR-25-3p	KLF4			
HDAC1	miR-25-3p	RECK			
HDAC1	miR-25-3p	TP53			
HDAC2	miR-25-3p	CDH1			
HDAC2	miR-25-3p	CDKN1A			
HDAC2	miR-25-3p	KLF4			
JUND	miR-25-3p	CDKN1A			
JUND	miR-25-3p	ERBB2			

JUND	miR-25-3p	MDM2			
MYC	miR-25-3p	TP53			
MYC	miR-25-3p	CDKN1A			
SP1	miR-25-3p	ATP2A2			
SP1	miR-25-3p	CDKN1A			
SP1	miR-25-3p	ERBB2			
SP1	miR-25-3p	MDM2			
SP1	miR-25-3p	PTEN			
SP1	miR-25-3p	RECK			
YY1	miR-25-3p	ERBB2			
YY1	miR-25-3p	SMAD7			
BRCA1	miR-25-3p	CDKN1A			
BRCA1	miR-25-3p	MDM2			
TCF3	miR-25-3p	CDH1			
TCF3	miR-25-3p	CDKN1A			
EP300	miR-25-3p	COL1A2			
HDAC1	miR-25-3p	COL1A2			
HDAC2	miR-25-3p	COL1A2			
JUND	miR-25-3p	ITGAV			
MYC	miR-25-3p	FMR1			
NRF1	miR-25-3p	FMR1			
RFX5	miR-25-3p	COL1A2			
SP1	miR-25-3p	FMR1			
SP1	miR-25-3p	ITGA5			
USF2	miR-25-3p	FMR1			
YY1	miR-25-3p	COL1A2			
TP53	miR-185-5p	AQP3			
TP53	miR-185-5p	DNMT1			
TP53	miR-185-5p	IGF1R			
TP53	miR-185-5p	VEGFA			
FOS	miR-185-5p	SMAD7			
MYC	miR-185-5p	VEGFA			
STAT3	miR-185-5p	AR			
STAT3	miR-185-5p	DNMT1			
STAT3	miR-185-5p	EPAS1			
STAT3	miR-185-5p	TGFB1			
STAT6	miR-185-5p	ALOX12			
STAT6	miR-185-5p	COL1A1			
STAT6	miR-185-5p	RHOA			
YY1	miR-185-5p	EPAS1			
YY1	miR-185-5p	SMAD7			
CTCF	miR-185-5p	APP			
ETS2	miR-185-5p	APP			
MYC	miR-185-5p	CCND2			
STAT3	miR-185-5p	CCND2			
USF1	miR-185-5p	SF1			

Supplementary Table S2

GO-term	Count	P-Value	Benjamini	Genes
GO:0045449~regulation of transcription	348	7.81E-79	8.83E-76	STAT5A, REST, MXI1, CTNNB1, PGR, APP, FLI1, SIN3A, PATZ1, TWIST2, TWIST1, PHOX2B, RCOR1, YY1, HNF4G, PROX1, FRY, MAPK1, RAB18, JUN, VEGFA, ZNF384, TGIF1, PRDM1, SUV420H1, TAF9B, AFAP1L2, MYT1, MYBL2, NR1H2, TAL1, LHX2, HOXA10, NKX2-1, TCF4, TCF3, NR1H3, KLF5, IKZF4, KLF6, ESRRA, SMAD7, KLF13, CREBBP, KLF11, ESRRG, RYBP, SMAD4, SMAD3, SKI, SMAD2, ABCG4, USF1, ABCG1, USF2, NR1I2, RNF4, ETS1, TRPS1, ETS2, RNF2, MZF1, SMURF2, KLF2, RBPJ, NCOR1, ZNF385A, TCF12, KLF4, RERE, BACH1, CCNT2, COPS2, PPARA, ELF1, ZBTB33, FOXA2, PPARG, EZH2, MITF, SPI1, TP63, NFKB1, ZEB2, ZEB1, NFKB2, RFXANK, YBX1, ZBTB37, GLI1, NOD2, HSF1, HSF2, NR2F2, MYB, MYC, AR, CTBP2, ZNF287, ZNF543, TP53, SF1, TLE3, CCNC, ZNF143, MBD1, MYCN, MAP3K10, FOXC1, CLOCK, TBX19, ETV7, MTDH, PML, FHL2, ZNF652, POLR2A, MYCBP2, STAT6, STAT4, BCL11B, PER2, GATAD2B, BCL3, ETV1, BCL6, AATF, THAP1, ETV6, BAZ2A, ZSCAN29, FOXD1, ETV4, MLLT3, ZNF263, TBX5, TRIM28, AFF4, STAT1, STAT3, STAT2, ATRX, YWHAH, UBTF, MAPK14, BNC1, ZNF460, PBX1, PBX3, PBX2, NR5A1, THRA, IL16, LMO3, CBX3, TBP, FOXO3, TCEAL1, TGFB1, GATA1, KDM1A, GATA2, HOXC9, MIER1, GATA3, GATA4, CIITA, RELA, GABPA, MTA3, SIX3, MTA1, SIX2, SIX5, ARNTL, HDAC11, ARID1B, GRHL1, NR0B1, TRERF1, ELL2, ZNF2, SUZ12, EP300, HIF1A, HOXD3, SIX1, TFAP2A, RNF20, HMGB1, HDGF, SOX4, ELK1, TRRAP, SOX9, LIN28A, SRF, ATF1, WT1, ATF2, SRY, VDR, MEIS2, SMARCB1, ELK4, TEAD4, JUND, NKX3-2, BHLHE40, RUNX1, RUNX2, EWSR1, RUNX3, SETDB1, SREBF1, TAF1, MAFF, EPAS1, RFX5, MAFB, TAF7, FOXA1, NR4A2, ARID3A, TEAD1, WHSC1, MAFK, BRCA1, FOXP2, SREBF2, HDAC5, CDKN1C, HDAC4, HDAC3, NRF1, HDAC2, ATF3, HDAC1, EAF1, GTF2F1, EBF1, RFX1, HIVEP2, DNMT1, MAFA, ABL1, HDAC9, HDAC6, E2F1, BMI1, E2F2, E2F3, FOSL2, E2F4, E2F6, FOXK2, PAX6, PAX5, RHOQ, CTCF, ZKSCAN1, HDX, CBFA2T3, PAX2, PDCD4, TMF1, PCGF5, SAP30, FOS, MAX, MAZ, PAX9, RHOA, ATP8B1, NFIL3, KDM5A, KDM5B, FOSL1, EGR1, TBL1XR1, SOX10, NANOG, EGR2, ZNF280C, SOX13, ESR1, BANP, RB1, HMGA1, UBN1, BTG2, ZMIZ1, HIPK3, HIPK2, MDM2, LCOR, CUX1, DPF2, GLIS3, BCLAF1, FOXM1, HCFC1, NFYC, NFYB, NFYA, TCF7L2, HIC2, NPAS2, CHD7, ERCC6, HAND2, POU2F2, CEBPZ, CHD2, CHD1, NFATC3, CHD4, NFATC1, MAF, KAT2A, ZBTB7A, ERG, FOXL2, CEBPB, KAT2B, FOXL1, TNFSF4, VHL, CEBPD, CREB1, SIRT6, SIRT7, SIRT1, ATXN1, IRF9, GMCL1, SP1, IRF6, SP2, SP3, PHB2, SP4, IRF1, IRF3, KDM4A, IRF4, NFIC, NFIB
GO:0009891~regulation of biosynthetic process	155	1.44E-58	4.43E-56	THRA, STAT5A, EDN1, EIF5A, TBP, FOXO3, TGFB1, CTNNB1, GATA2, APP, GATA4, CIITA, RELA, SIX3, SIX2, ARNTL, ARID1B, PROX1, TRERF1, MAPK1, EP300, HIF1A, JUN, SIX1, VEGFA, RNF20, ELK1, SOX4, AFAP1L2, SOX9, SRF, WT1, NR1H2, MEIS2, SMARCB1, TEAD4, NKX2-1, TCF4, RUNX1, TRAF6, RUNX2, TCF3, NR1H3, KLF5, SREBF1, KLF6, TAF1, ESRRA, EPAS1, MAFB, KLF13, CREBBP, FOXA1, TAF7, SMAD4, ARPP19, NR4A2, ESRRG, TEAD1, SMAD3, SMAD2, USF1, ABCG4, BRCA1, ABCG1, USF2, SREBF2, HDAC5, HDAC4, HDAC2, RNF4, HDAC1, ETS1, ETS2, GTF2F1, EBF1, MAFA, KLF2, KLF4, HDAC6, E2F1, PPARA, ELF1, E2F3, FOXA2, MITF, PPARG, SPI1, PAX6, RHOQ, TP63, CTCF, NFKB1, ZEB1, PAX2, GLI1, FOS, NOD2, PAX9, NR2F2, KDM5A, FOSL1, MYC, EGR1, SOX10, TBL1XR1, AR, EGR2, ZNF287, TP53, RB1, HMGA1, ZMIZ1, HIPK2, FOXC1, CLOCK, TBX19, GLIS3, FHL2, KITLG, NFYC, NFYB, NFYA, TCF7L2, STAT6, IGF1R, NPAS2, HAND2, BCL11B, BCL3, AATF, NFATC3, ETV4, MAF, FOXL2, CEBPB, VHL, CREB1, TBX5, TRIM28, NPR1, SIRT7, STAT3, ATXN1, YWHAH, SP1, IRF6, MAPK14, IRF1, PBX1, IRF4, NFIC, PBX2, NFIB, NR5A1
GO:0051173~positive regulation of nitrogen compound metabolic process	149	2.38E-58	6.72E-56	THRA, STAT5A, EDN1, TBP, FOXO3, TGFB1, CTNNB1, GATA2, APP, GATA4, CIITA, RELA, SIX3, SIX2, ARNTL, ARID1B, PROX1, TRERF1, MAPK1, EP300, HIF1A, JUN, SIX1, VEGFA, RNF20, ELK1, SOX4, AFAP1L2, SOX9, SRF, WT1, NR1H2, MEIS2, SMARCB1, TEAD4, NKX2-1, TCF4, RUNX1, RUNX2, TCF3, NR1H3, KLF5, SREBF1, KLF6, TAF1, ESRRA, EPAS1, MAFB, KLF13, CREBBP, FOXA1, TAF7, SMAD4, NR4A2, ESRRG, TEAD1, SMAD3, SMAD2, USF1, BRCA1, USF2, SREBF2, HDAC5, HDAC4, HDAC2, RNF4, HDAC1, ETS1, ETS2, GTF2F1, EBF1, MAFA, KLF2, KLF4, E2F1, PPARA, ELF1, E2F3, FOXA2, MITF, PPARG, PAX6, SPI1, RHOQ, TP63, CTCF, NFKB1, ZEB1, PAX2, GLI1, FOS, PAX9, NR2F2, KDM5A, FOSL1, MYC, EGR1, SOX10, TBL1XR1, AR, EGR2, ZNF287, TP53, RB1, HMGA1, ZMIZ1, HIPK2, FOXC1, CLOCK, TBX19, GLIS3, FHL2, KITLG, NFYC, NFYB, NFYA, TCF7L2, STAT6, IGF1R, NPAS2, ERCC6, HAND2, BCL11B, BCL3, AATF, NFATC3, ETV4, MAF, FOXL2, CEBPB, VHL, CREB1, TBX5, TRIM28, NPR1, SIRT7, STAT3, ATXN1, YWHAH, SP1, IRF6, MAPK14, IRF1, PBX1, IRF4, NFIC, PBX2, NFIB, NR5A1
GO:0043009~chordate embryonic development	56	6.93E-15	6.48E-13	GNA13, FOXA2, EDN1, PAX6, ZEB2, ZEB1, YBX1, KDM1A, GATA2, HOXC9, GRIN2B, HSF1, GATA4, TWIST1, SOX10, AR, ADAM10, GABPA, TP53, SIX2, PROX1, BCL2L11, HIF1A, EP300, HOXD3, ZMIZ1, SIX1, TGIF1, TFAP2A, FOXC1, COL1A1, PRDM1, SYVN1, DSCAML1, CDH1, SRF, TCF7L2, CHD7, HAND2, TEAD4, NKX3-2, TRAF6, KAT2A, MAFF, CEBPB, EPAS1, SMAD2, ATM, BRCA1, TJP1, SP1, TSC1, SP3, MAPK8IP3, PBX1, RBPJ
GO:0043067~regulation of programmed cell death	92	7.80E-13	6.45E-11	STAT5A, MITF, EIF5A, TP63, NFKB1, FOXO3, SGMS1, PAX2, PTEN, TGFB1, BAK1, PAK7, NOD2, APP, RHOA, FAS, CASP2, MYC, FOSL1, TWIST2, BCL2L14, RELA, ESR1, TP53, MBD4, IL6R, BCL2L11, MAPK1, BTG2, IGF2R, HIPK3, JUN, HIPK2, VEGFA, MAP3K10, NAIF1, FOXC1, SEMA4D, TNFAIP3, MYO18A, FAIM2, ALOX12, DPF2, HMGB1, BCLAF1, YWHAZ, SYVN1, MCL1, ERBB3, ERBB2, TAF9B, PML, SOX4, CDH1, SOX9, TCF7L2, SERINC3, VDR, IGF1R, ERCC6, BCL2, BCL11B, RAC1, NKX3-2, BCL3, BCL6, AATF, TRAF6, BMF, RUNX3, RASA1, FOXL2, CEBPB, VHL, TBX5, CREB1, NR4A2, SMAD3, STAT1, SIRT1, ATM, BRCA1, CDKN1A, HDAC3, NUPR1, HDAC1, CASP14, ETS1, ABL1, TIAF1, TP53INP1, HDAC6

GO:0048534~hemopoietic or lymphoid organ development	45	2.00E-12	1.41E-10	BMI1, STAT5A, PPARG, PML, KITLG, SOX4, NFKB2, TGFB1, CTNNB1, CDC42, TAL1, BAK1, CHD7, BCL11B, BCL2, NKX3-2, BCL3, PATZ1, BCL6, FAS, RUNX1, TRAF6, TCF3, EGR1, KLF6, LYN, EPAS1, TTC7A, TAZ, TP53, CDK6, RB1, BCL2L11, HDAC5, HDAC4, CRKL, SP1, SP3, VEGFA, SIX1, IRF1, PBX1, IRF4, HDAC9, ADD2
GO:0035295~tube development	41	1.97E-12	1.42E-10	GNA13, NRP1, FOXA2, EDN1, TP63, ZEB2, PAX2, SRF, GJA5, CTNNB1, GLI1, HAND2, BCL2, GATA4, NKX2-1, TRAF6, TWIST1, LIPA, EPAS1, TBX5, FOXA1, SMAD4, SMAD2, PROX1, BCL2L11, FOXP2, MYCN, EP300, SP1, TSC1, SP3, VEGFA, SIX1, TGIF1, TFAP2A, MAPK8IP3, PBX1, FOXC1, CUX1, NFIB, BMPR1A
GO:0009725~response to hormone stimulus	54	6.70E-12	4.54E-10	PPARA, THRA, STAT5A, PPARG, RHOQ, AQP1, PTEN, LATS2, TGFB1, CTNNB1, FOS, PRKAR2A, EIF4EBP1, EIF4EBP2, GATA3, FAS, FOSL1, AR, EGR2, LYN, RELA, ESR1, IL6R, STXBP4, NR0B1, MAPK1, EP300, BTG2, CCND2, COL1A1, ERBB3, ERBB2, FHL2, TAC1, SRF, IGF1R, BCL2, MSI1, PIK3R3, KAT2B, MAT2A, MAP1B, STAT1, APPL1, USF1, ABCG1, BRCA1, STAT3, SREBF2, HDAC5, CDKN1A, TSC1, HDAC9, PARP1
GO:0002520~immune system development	45	1.60E-11	1.03E-09	BMI1, STAT5A, PPARG, PML, KITLG, SOX4, NFKB2, TGFB1, CTNNB1, CDC42, TAL1, BAK1, CHD7, BCL11B, BCL2, NKX3-2, BCL3, PATZ1, BCL6, FAS, RUNX1, TRAF6, TCF3, EGR1, KLF6, LYN, EPAS1, TTC7A, TAZ, TP53, CDK6, RB1, BCL2L11, HDAC5, HDAC4, CRKL, SP1, SP3, VEGFA, SIX1, IRF1, PBX1, IRF4, HDAC9, ADD2
GO:0007423~sensory organ development	40	3.03E-11	1.84E-09	ERBB3, ERBB2, MITF, EDN1, PAX6, NFYC, ZEB1, PAX2, WT1, TGFB1, EPHB2, CTNNB1, BAK1, MEIS2, CHD7, PVRL1, GRIN2B, BCL11B, BCL2, NKX3-2, MYC, MAF, FOXL2, MAFB, YY1, SIX3, SIX2, SIX5, PROX1, STAT3, BCL2L11, FOXP2, SP1, SP3, VEGFA, SIX1, TGIF1, FOXC1, CUX1, KLF4
GO:0048732~gland development	30	3.47E-11	2.03E-09	ERBB3, STAT5A, ERBB2, PAX6, HK2, TP63, CDH1, TGFB1, GLI1, PGR, GATA2, KDM1A, IGF1R, AGPAT6, BCL2, NKX2-1, AR, CREB1, FOXA1, NR0B1, USF2, BCL2L11, CRKL, IRF6, HOXD3, SIX1, PBX1, FOXC1, TBX19, NR5A1
GO:0030097~hemopoiesis	40	7.78E-11	4.40E-09	BMI1, STAT5A, PPARG, PML, KITLG, SOX4, TGFB1, CTNNB1, CDC42, TAL1, BAK1, CHD7, BCL11B, BCL2, BCL3, PATZ1, BCL6, FAS, RUNX1, TRAF6, TCF3, EGR1, KLF6, EPAS1, LYN, TTC7A, TAZ, TP53, CDK6, RB1, HDAC5, HDAC4, SP1, SP3, VEGFA, IRF1, PBX1, IRF4, HDAC9, ADD2
GO:0045165~cell fate commitment	29	3.71E-10	2.00E-08	MCL1, FOXA2, PPARG, MITF, PAX6, DSCAML1, SOX9, PAX2, TCF7L2, CTNNB1, CDC42, GATA2, TAL1, BCL2, TEAD4, NKX2-1, NR2F2, TCF3, RUNX2, FOXA1, SIX3, SMAD4, TP53, SMAD2, PROX1, SIX1, PRDM1, KLF4, TBX19
GO:0043010~camera-type eye development	25	6.79E-10	3.60E-08	MAF, FOXL2, YY1, MITF, SIX3, PAX6, NFYC, SIX5, ZEB1, PAX2, PROX1, WT1, CTNNB1, EPHB2, FOXP2, BAK1, CHD7, SP1, PVRL1, SP3, BCL11B, VEGFA, TGIF1, FOXC1, KLF4
GO:0009952~anterior/posterior pattern formation	28	2.11E-09	1.05E-07	FOXA2, PAX6, ZEB2, CTNNB1, HOXC9, GATA4, HOXA10, NR2F2, LFNG, MLLT3, KAT2A, ARC, YY1, SIX3, SMAD4, CYP26A1, SMAD2, ATM, CRKL, EP300, BTG2, RNF2, HOXD3, HIPK2, PBX1, RBPJ, PBX3, BMPR1A
GO:0006476~protein amino acid deacetylation	12	2.55E-09	1.26E-07	HDAC5, HDAC4, HDAC3, HDAC2, HDAC1, SIRT6, SIRT7, HDAC11, HDAC9, BAZ2A, SIRT1, HDAC6
GO:0006325~chromatin organization	49	5.60E-09	2.67E-07	BMI1, HP1BP3, EZH2, CBX3, CTCF, KDM1A, H2AFZ, KDM5A, KDM5B, TBL1XR1, RCOR1, BANP, RB1, ARID1B, HDAC11, HMGA1, UBN1, SUZ12, EP300, RNF20, SUV420H1, HMGB1, TRRAP, CHD7, SMARCB1, CHD2, CHD1, BAZ2A, CHD4, SETDB1, KAT2A, KAT2B, CREBBP, WHSC1, SIRT1, HDAC5, HDAC4, HDAC3, HDAC2, HDAC1, RNF2, MSL1, DNMT1, KDM4A, IRF4, HDAC9, NCOR1, RERE, HDAC6
GO:0045637~regulation of myeloid cell differentiation	19	9.36E-09	4.35E-07	ZBTB7A, ESRRA, MAFB, STAT5A, MITF, SPI1, KITLG, CDK6, RB1, FOXO3, CTNNB1, TAL1, HIF1A, ETS1, MAPK14, JUN, FAS, RUNX1, TOB2
GO:0050767~regulation of neurogenesis	29	2.37E-08	1.07E-06	NRP1, FOXA2, PPARG, PAX6, REST, CDH4, TGFB1, TTC3, EPHB2, BCL2, RHOA, SEMA3A, PHOX2B, LYN, RELA, FOXA1, MAP1B, TP53, NTRK3, YWHAH, SEMA4F, CCND2, HOXD3, SIX1, TGIF1, PBX1, SEMA4D, DBN1, BMPR1A
GO:0032868~response to insulin stimulus	22	2.75E-08	1.22E-06	PPARA, AR, KAT2B, EGR2, LYN, ERBB3, RELA, PPARG, RHOQ, STXBP4, APPL1, STAT1, USF1, HDAC5, IGF1R, EIF4EBP1, EIF4EBP2, TSC1, CCND2, PARP1, HDAC9, PIK3R3
GO:0016575~histone deacetylation	10	3.97E-08	1.75E-06	HDAC5, HDAC4, HDAC3, HDAC2, HDAC1, HDAC11, HDAC9, BAZ2A, SIRT1, HDAC6
GO:0051960~regulation of nervous system development	31	4.55E-08	1.98E-06	NRP1, FOXA2, PPARG, PAX6, REST, CDH4, TGFB1, TTC3, EPHB2, GLI1, PVRL1, BCL2, RHOA, SEMA3A, PHOX2B, LYN, RELA, MAP1B, FOXA1, TP53, NTRK3, YWHAH, SEMA4F, CCND2, HOXD3, SIX1, TGIF1, PBX1, SEMA4D, DBN1, BMPR1A
GO:0002521~leukocyte differentiation	25	4.68E-08	2.01E-06	EGR1, KLF6, STAT5A, PPARG, TP53, SOX4, TGFB1, CTNNB1, HDAC5, HDAC4, CDC42, BAK1, CHD7, SP3, BCL2, BCL11B, IRF1, BCL3, PATZ1, BCL6, FAS, IRF4, TRAF6, HDAC9, TCF3
GO:0001501~skeletal system development	42	5.31E-08	2.25E-06	CYP24A1, THRA, EDN1, FHL2, DSCAML1, TP63, ZEB1, SOX9, GJA5, TGFB1, CTNNB1, GLI1, VDR, HOXC9, CHD7, BCL2, TEAD4, JUND, NKX3-2, HOXA10, TRAF6, RUNX2, MYC, TWIST1, ESRRA, SMAD3, SIX2, HDAC4, SP1, MAPK14, ETS2, TRPS1, SP3, HOXD3, SIX1, COL1A2, TFAP2A, PBX1, FOXC1, COL1A1, PLEKHA1, BMPR1A
GO:0032989~cellular component morphogenesis	47	1.91E-07	7.20E-06	E2F4, NRP1, ERBB3, ERBB2, PAX6, DSCAML1, SOX9, PAX2, CDH4, LLGL1, TGFB1, MYCBP2, EPHB2, CTNNB1, CDC42, IGF1R, BAK1, APP, PVRL1, LHX2, BCL11B, BCL2, BAI1, RAC1, RHOA, NKX2-1, BCL6, SEMA3A, ETV4, RUNX3, NFATC1, NOX4, EGR2, LIPA, VHL, CREB1, MAP1B, TRIM28, LIFR, NR4A2, PROX1, MFN1, HIF1A, SEMA4F, MAPK8IP3, MAP7, SLITRK6

GO:0030098~lymphocyte differentiation	21	2.30E-07	8.29E-06	EGR1, KLF6, STAT5A, TP53, SOX4, TGFB1, CTNNB1, HDAC5, HDAC4, BAK1, CHD7, SP3, BCL2, BCL11B, IRF1, BCL3, PATZ1, BCL6, FAS, HDAC9, TCF3
GO:0030278~regulation of ossification	18	3.29E-07	1.17E-05	ESRRA, EGR2, SMAD3, CDK6, SKI, IL6R, SOX9, TGFB1, CTNNB1, BCL2, JUND, PBX1, SMURF1, RUNX2, TWIST2, TOB2, BMPR1A, TOB1
GO:0060541~respiratory system development	21	5.13E-07	1.76E-05	LIPA, FOXA2, EPAS1, TBX5, FOXA1, SMAD2, PROX1, GLI1, CTNNB1, MYCN, FOXP2, EP300, CHD7, SP1, SP3, VEGFA, MAPK8IP3, NKX2-1, CUX1, NFIB, BMPR1A
GO:0030324~lung development	20	5.56E-07	1.87E-05	LIPA, FOXA2, EPAS1, TBX5, FOXA1, SMAD2, PROX1, GLI1, CTNNB1, MYCN, FOXP2, EP300, SP1, SP3, VEGFA, MAPK8IP3, NKX2-1, CUX1, NFIB, BMPR1A
GO:0007507~heart development	31	5.77E-07	1.92E-05	NRP1, ERBB3, ERBB2, EDN1, SOX4, SOX9, SRF, GJA5, PTEN, CTNNB1, CHD7, HAND2, GATA4, NFATC3, NFATC1, SMAD7, TBX5, TAZ, TEAD1, SMAD2, PROX1, ATM, HDAC5, CRKL, EP300, TSC1, ZMIZ1, FOXC1, RBPJ, HDAC9, BMPR1A
GO:0042493~response to drug	31	6.38E-07	2.08E-05	YWHAZ, ERBB3, ERBB2, PPARG, CDH1, PTEN, TGFB1, FOS, BCL2, FOSL1, SLC8A1, LYN, MAT2A, CREB1, RELA, MAP1B, ATP1A3, PAPD7, TP53, STAT1, STAT3, SREBF2, ABCG2, HDAC4, CDKN1A, HDAC3, HDAC2, EP300, JUN, ABCC1, SLC9A1
GO:0045646~regulation of erythrocyte differentiation	9	1.29E-06	4.01E-05	TAL1, HIF1A, ETS1, MAFB, MAPK14, STAT5A, SPI1, CDK6, FOXO3
GO:0010869~regulation of receptor biosynthetic process	7	1.32E-06	4.04E-05	PPARA, HDAC2, HDAC1, ITGAV, PPARG, ITGB3, HDAC6
GO:0030182~neuron differentiation	48	1.31E-06	4.04E-05	NRP1, FOXA2, ERBB3, ERBB2, PAX6, DSCAML1, CDH1, PRKG1, PAX2, SRF, PTEN, CDH4, MYCBP2, EPHB2, CDC42, IGF1R, GATA2, APP, PVRL1, DYNLL2, LHX2, BCL11B, BCL2, BAI1, RAC1, NKX2-1, SEMA3A, ETV4, RUNX3, CEBPB, EGR2, CREB1, MDGA2, MAP1B, FOXA1, LIFR, SMAD4, NR4A2, STAT3, NTRK3, CDKN1C, BTG2, SEMA4F, VEGFA, MAPK8IP3, PBX3, SLITRK6, CUX1
GO:0007409~axonogenesis	28	2.00E-06	5.90E-05	NRP1, ERBB3, ERBB2, PAX6, DSCAML1, PAX2, CDH4, MYCBP2, EPHB2, IGF1R, APP, PVRL1, BCL11B, BCL2, LHX2, RAC1, BAI1, NKX2-1, SEMA3A, ETV4, RUNX3, EGR2, CREB1, MAP1B, NR4A2, SEMA4F, MAPK8IP3, SLITRK6
GO:0034097~response to cytokine stimulus	17	2.06E-06	6.02E-05	CIITA, ADAM10, MCL1, RELA, LIFR, PML, IL6R, STAT1, SRF, STAT3, CTNNB1, STAT6, HDAC4, FOS, BCL2, JUN, FOSL1
GO:0007530~sex determination	9	2.13E-06	6.16E-05	IGF1R, SRY, FOXL2, AR, SF1, SOX9, NR0B1, WT1, NR5A1
GO:0010883~regulation of lipid storage	9	5.22E-06	1.38E-04	NR1H2, PPARA, ITGAV, PPARG, NFKB1, SCARB1, ITGB3, ABCG1, NR1H3
GO:0030334~regulation of cell migration	25	5.66E-06	1.49E-04	GNA13, EDN1, PAX6, TAC1, ITGB3, PTEN, TGFB1, IGF1R, MIA3, BCL2, RAC1, SCARB1, ADAM10, SMAD7, TBX5, SMAD3, IL6R, HDAC5, MAPK1, HIF1A, DLL4, VEGFA, HDAC9, RNF20, HDAC6
GO:0009628~response to abiotic stimulus	41	5.97E-06	1.56E-04	TG, PPARG, PML, TAC1, TGFB1, FOS, SNN, BAK1, APP, ERCC6, HSF1, GRIN2B, BCL2, RAC1, BCL3, NKX2-1, FOSL1, MYC, LYN, MAT2A, RELA, MAP1B, TP53, ATP1A3, MBD4, IL6R, STAT1, USF1, BRCA1, ATM, FOXP2, ATXN1, CDKN1A, HDAC2, XPC, HSPB6, BTG2, JUN, COL1A1, CLOCK, SLC9A1
GO:0048608~reproductive structure development	21	6.22E-06	1.61E-04	FOXL2, AR, STAT5A, FOXA1, SF1, TP63, FOXO3, NR0B1, SOX9, SIRT1, BCL2L11, WT1, PGR, BAK1, CCND2, BCL2, VEGFA, NKX2-1, FOXC1, LFNG, NR5A1
GO:0048667~cell morphogenesis involved in neuron differentiation	28	9.19E-06	2.26E-04	NRP1, ERBB3, ERBB2, PAX6, DSCAML1, PAX2, CDH4, MYCBP2, EPHB2, IGF1R, APP, PVRL1, BCL11B, BCL2, LHX2, RAC1, BAI1, NKX2-1, SEMA3A, ETV4, RUNX3, EGR2, CREB1, MAP1B, NR4A2, SEMA4F, MAPK8IP3, SLITRK6
GO:0060485~mesenchyme development	13	9.52E-06	2.32E-04	HIF1A, HAND2, BCL2, TRIM28, EDN1, CYP26A1, KITLG, ZEB2, FOXC1, SOX9, TGFB1, CTNNB1, NFATC1
GO:0030900~brain development	23	1.01E-05	2.45E-04	E2F1, CREB1, SIX3, PAX6, CDH1, ZEB2, PRKG1, NR0B1, GLI1, CTNNB1, FOXP2, ATRX, KDM1A, GATA2, APP, TSC1, LHX2, RAC1, MAPK8IP3, NKX2-1, NR2F2, TBX19, NFIB
GO:0018205~peptidyl-lysine modification	8	1.13E-05	2.73E-04	HDAC4, EP300, KAT2B, CREBBP, EIF5A, HDAC9, SIRT1, HDAC6
GO:0010888~negative regulation of lipid storage	7	1.27E-05	3.02E-04	NR1H2, PPARA, ITGAV, PPARG, ITGB3, ABCG1, NR1H3
GO:0010745~negative regulation of foam cell differentiation	7	1.27E-05	3.02E-04	NR1H2, PPARA, ITGAV, PPARG, ITGB3, ABCG1, NR1H3
GO:0008629~induction of apoptosis by intracellular signals	13	1.44E-05	3.39E-04	CDKN1A, ERCC6, NUPR1, HIPK2, PML, TP53, BCL3, TP63, MBD4, ABL1, MYC, BRCA1, ATM
GO:0060429~epithelium development	29	1.52E-05	3.55E-04	E2F4, FOXA2, PPARG, PAX6, TP63, ZEB2, PAX2, WT1, CTNNB1, PGR, AGPAT6, BCL2, MSI1, NKX2-1, TRAF6, NR2F2, TWIST1, TBX5, FOXA1, SMAD4, PROX1, TSC1, IRF6, JUN, VEGFA, SIX1, TGIF1, TFAP2A, PBX1
GO:0006913~nucleocytoplasmic transport	23	1.54E-05	3.58E-04	SMG7, SIX3, TP53, PML, EIF5A, SIX2, ARNTL, TGFB1, ATXN1, MAPK1, CAMK4, TSC1, RAE1, IPO7, JUN, TRPS1, IPO5, ZNF384, BCL3, BCL6, SMURF1, RERE, TOB1

GO:0030217~T cell differentiation	14	2.14E-05	4.71E-04	EGR1, STAT5A, TP53, SOX4, TGFB1, CTNNB1, CHD7, BCL11B, SP3, BCL2, IRF1, BCL3, PATZ1, FAS
GO:0048771~tissue remodeling	13	2.13E-05	4.75E-04	LIPA, EPAS1, NCDN, ERBB3, ERBB2, MITF, TGFB1, CTNNB1, BAK1, HIF1A, RAC1, VEGFA, FOXC1
GO:0030522~intracellular receptor-mediated signaling pathway	15	2.38E-05	5.19E-04	CYP24A1, AR, TAF7, ESR1, PML, FHL2, CYP26A1, RB1, BRCA1, CTNNB1, NR1H2, PGR, VDR, YWHAH, RNF4
GO:0048469~cell maturation	15	2.38E-05	5.19E-04	SOX10, EPAS1, FOXA1, PPARG, NR4A2, FOXO3, CTNNB1, PGR, CDKN1C, GATA2, APP, AGPAT6, VEGFA, RUNX2, RUNX3
GO:0048666~neuron development	37	2.83E-05	6.07E-04	NRP1, ERBB3, ERBB2, PAX6, DSCAML1, CDH1, PRKG1, PAX2, SRF, PTEN, CDH4, MYCBP2, EPHB2, IGF1R, APP, PVRL1, DYNLL2, BCL11B, BCL2, LHX2, BAI1, RAC1, NKX2-1, SEMA3A, ETV4, RUNX3, EGR2, CREB1, MAP1B, NR4A2, LIFR, CDKN1C, SEMA4F, VEGFA, MAPK8IP3, PBX3, SLITRK6
GO:0045639~positive regulation of myeloid cell differentiation	10	3.00E-05	6.39E-04	TAL1, HIF1A, ETS1, MAPK14, JUN, STAT5A, KITLG, RB1, FOXO3, RUNX1
GO:0040008~regulation of growth	37	3.27E-05	6.92E-04	NRP1, STAT5A, FOXM1, PPARG, TAF9B, PML, TP63, CDH4, TGFB1, AGPAT6, APP, CHD7, HSF1, BCL2, GATA4, CAMK2D, BCL6, SEMA3A, ADAM10, TBX5, CREB1, MAP1B, SMAD4, TP53, SMAD3, NPR1, RB1, PROX1, BCL2L11, STAT3, NTRK3, CDKN1A, EP300, SEMA4F, FOXC1, PARP1, ALOX12
GO:0007167~enzyme linked receptor protein signaling pathway	37	3.49E-05	7.35E-04	NRP1, ERBB3, STAT5A, ERBB2, PML, RHOQ, PTEN, TGFB1, EPHB2, IGF1R, FOS, EIF4EBP1, EIF4EBP2, PIK3R3, PTPRE, SMAD7, CREB1, SMAD4, LIFR, SMAD3, RAF1, SKI, SMAD2, STXBP4, APPL1, STAT3, NTRK3, JUN, HIPK2, VEGFA, COL1A2, FOXC1, SMURF2, SMURF1, PLEKHA1, TOB1, BMPR1A
GO:0022604~regulation of cell morphogenesis	20	3.90E-05	8.16E-04	GNA13, NRP1, SMAD7, MAP1B, SMAD4, SMAD3, RHOQ, SMAD2, CDH4, TTC3, TGFB1, EPHB2, NTRK3, YWHAH, SEMA4F, VEGFA, RHOA, SEMA3A, SEMA4D, RASA1
GO:0046660~female sex differentiation	14	4.17E-05	8.67E-04	FOXL2, STAT5A, TP63, FOXO3, SIRT1, PGR, CHD7, CCND2, BCL2, VEGFA, NKX2-1, FOXC1, LFNG, NR5A1
GO:0046545~development of primary female sexual characteristics	14	4.17E-05	8.67E-04	FOXL2, STAT5A, TP63, FOXO3, SIRT1, PGR, CHD7, CCND2, BCL2, VEGFA, NKX2-1, FOXC1, LFNG, NR5A1
GO:0048592~eye morphogenesis	14	4.17E-05	8.67E-04	FOXL2, YY1, SIX3, PAX6, ZEB1, PROX1, STAT3, EPHB2, CTNNB1, BAK1, PVRL1, SP1, SP3, VEGFA
GO:0014070~response to organic cyclic substance	19	4.40E-05	9.09E-04	LYN, RELA, PPARG, TAF9B, TAC1, STAT1, PTEN, STAT3, TGFB1, CTNNB1, FOS, CDKN1A, HDAC2, BTG2, CCND2, JUN, BCL2, FOSL1, SLC9A1
GO:0048562~embryonic organ morphogenesis	20	4.83E-05	9.91E-04	FOXL2, MAFB, EDN1, DSCAML1, SIX2, ZEB1, PAX2, TCF7L2, PROX1, GLI1, CHD7, HOXC9, SP1, HOXD3, SP3, SIX1, GATA4, NKX3-2, TFAP2A, MYC
GO:0045321~leukocyte activation	29	4.93E-05	0.001001209	YWHAZ, STAT5A, EDN1, SOX4, TGFB1, CTNNB1, BAK1, CHD7, BCL11B, BCL2, BCL3, PATZ1, BCL6, FAS, TRAF6, TCF3, EGR1, KLF6, ADAM10, TNFSF4, LYN, TP53, SMAD3, HDAC5, HDAC4, SP3, IRF1, IRF4, HDAC9
GO:0045667~regulation of osteoblast differentiation	11	4.92E-05	0.00100332	ESRRA, JUND, SMAD3, CDK6, SKI, IL6R, RUNX2, TWIST2, TOB1, CTNNB1, BMPR1A
GO:0009636~response to toxin	13	5.25E-05	0.001053515	BAK1, FOS, CDKN1A, ERCC6, NUPR1, LYN, BCL2, MAP1B, NR4A2, CDH1, FAS, SRF, HDAC6
GO:0021700~developmental maturation	17	5.32E-05	0.001060047	RECK, SOX10, EPAS1, FOXA1, MAP1B, PPARG, NR4A2, FOXO3, CTNNB1, PGR, CDKN1C, GATA2, APP, AGPAT6, VEGFA, RUNX2, RUNX3
GO:0001817~regulation of cytokine production	24	5.41E-05	0.001072816	ELF1, TNFSF4, CEBPB, ZNF287, CREB1, STAT5A, RELA, PPARG, SMAD4, SMAD3, AFAP1L2, NFKB1, IL6R, IGF2BP3, TGFB1, NOD2, HIF1A, HSF1, GATA4, IRF1, BCL3, BCL6, IRF4, TRAF6
GO:0008406~gonad development	18	5.49E-05	0.001080988	AR, FOXL2, STAT5A, SF1, FOXO3, SOX9, NR0B1, SIRT1, BCL2L11, WT1, PGR, CCND2, BCL2, VEGFA, NKX2-1, FOXC1, LFNG, NR5A1
GO:0033365~protein localization in organelle	21	5.67E-05	0.001110286	YWHAZ, SIX3, TP53, PAX6, PML, SIX2, ARNTL, TGFB1, MAPK1, SRPR, TOMM70A, IPO7, JUN, TRPS1, IPO5, PIKFYVE, BCL3, BCL6, RERE, TOB1, NR5A1
GO:0021983~pituitary gland development	9	5.79E-05	0.001127111	KDM1A, GATA2, CREB1, PAX6, NKX2-1, CDH1, NR0B1, TBX19, GLI1
GO:0048593~camera-type eye morphogenesis	11	6.07E-05	0.001175526	BAK1, SP1, PVRL1, YY1, SP3, VEGFA, SIX3, ZEB1, PROX1, EPHB2, CTNNB1
GO:0022602~ovulation cycle process	13	6.22E-05	0.001196592	PGR, FOXL2, CCND2, STAT5A, BCL2, ERBB2, VEGFA, NKX2-1, FOXC1, FOXO3, SIRT1, LFNG, NR5A1
GO:0060537~muscle tissue development	19	6.81E-05	0.001296951	FOXL2, ERBB3, SMAD7, TBX5, ERBB2, TAZ, TP63, PTEN, PROX1, FOXP2, APP, EP300, TSC1, MAPK14, DNER, SIX1, RHOA, FOXC1, NR2F2
GO:0043433~negative regulation of transcription factor activity	11	7.45E-05	0.001410284	HDAC4, KDM1A, NOD2, THRA, TNFSF4, HDAC2, SMAD7, PBX1, NR0B1, PROX1, SIRT1

GO:0032880~regulation of protein localization	20	8.05E-05	0.001507101	TNFSF4, SMAD4, SMAD3, RHOQ, CDH1, MXI1, APPL1, SIRT1, TGFB1, UHMK1, MYCBP2, NOD2, WWP2, BCL2, RHOA, RAB14, RAB11A, BCL3, TRAF6, BMPR1A
GO:0021536~diencephalon development	10	8.04E-05	0.001513136	KDM1A, GATA2, CREB1, SIX3, PAX6, NKX2-1, CDH1, NR0B1, TBX19, GLI1
GO:0001763~morphogenesis of a branching structure	14	8.91E-05	0.00164105	GNA13, NRP1, FOXA2, FOXA1, EDN1, SMAD4, PAX2, TGFB1, MYCN, CTNNB1, BCL2, VEGFA, SIX1, PBX1
GO:0051154~negative regulation of striated muscle cell differentiation	5	9.53E-05	0.001745758	HDAC5, HDAC4, HDAC3, HDAC1, EZH2
GO:0010887~negative regulation of cholesterol storage	5	9.53E-05	0.001745758	NR1H2, PPARA, PPARG, ABCG1, NR1H3
GO:0060395~SMAD protein signal transduction	5	9.53E-05	0.001745758	FOS, JUN, HIPK2, SMAD4, SKI
GO:0048754~branching morphogenesis of a tube	13	1.01E-04	0.001832558	GNA13, NRP1, FOXA2, BCL2, VEGFA, EDN1, FOXA1, SIX1, SMAD4, PBX1, PAX2, CTNNB1, MYCN
GO:0007411~axon guidance	17	1.08E-04	0.001950126	NRP1, EGR2, ERBB2, PAX6, CDH4, EPHB2, MYCBP2, APP, PVRL1, SEMA4F, LHX2, RAC1, MAPK8IP3, NKX2-1, SEMA3A, RUNX3, ETV4
GO:0006606~protein import into nucleus	15	1.14E-04	0.002042459	SIX3, TP53, PML, SIX2, ARNTL, TGFB1, MAPK1, IPO7, JUN, TRPS1, IPO5, BCL3, BCL6, RERE, TOB1
GO:0014706~striated muscle tissue development	18	1.19E-04	0.00212152	FOXL2, ERBB3, SMAD7, TBX5, ERBB2, TAZ, PTEN, PROX1, FOXP2, APP, EP300, TSC1, MAPK14, DNER, SIX1, RHOA, FOXC1, NR2F2
GO:0007156~homophilic cell adhesion	19	1.26E-04	0.002217249	PCDHA6, PCDHA7, PCDHA8, PCDHA9, PCDHA2, PCDH11Y, PCDHA3, PCDH11X, PCDHA4, PCDH10, PCDHA5, DSCAML1, CDH1, PCDHA1, CDH4, PCDHAC2, PCDHAC1, CDH20, PVRL1, PCDHA10, DSC2, PCDHA11, PCDHA12, PCDHA13
GO:0030238~male sex determination	6	1.26E-04	0.002218357	IGF1R, SRY, AR, SF1, SOX9, NR0B1
GO:0010885~regulation of cholesterol storage	6	1.26E-04	0.002218357	NR1H2, PPARA, PPARG, SCARB1, ABCG1, NR1H3
GO:0010721~negative regulation of cell development	11	1.33E-04	0.002307743	NRP1, YWHAH, SEMA4F, SMAD7, RHOA, TP53, SEMA3A, TTC3, TGFB1, EPHB2, BMPR1A
GO:0030183~B cell differentiation	11	1.33E-04	0.002307743	HDAC5, HDAC4, KLF6, BAK1, BCL2, SP3, TP53, BCL3, BCL6, HDAC9, TCF3
GO:0040017~positive regulation of locomotion	16	1.35E-04	0.002330108	ADAM10, IL16, EDN1, SMAD3, TAC1, IL6R, TGFB1, IGF1R, MAPK1, MIA3, HIF1A, BCL2, VEGFA, SCARB1, HDAC9, HDAC6
GO:0042698~ovulation cycle	13	1.36E-04	0.002332426	PGR, FOXL2, CCND2, STAT5A, BCL2, ERBB2, VEGFA, NKX2-1, FOXC1, FOXO3, SIRT1, LFNG, NR5A1
GO:0032496~response to lipopolysaccharide	14	1.36E-04	0.002334538	TG, RELA, TAC1, IL6R, STAT1, FOS, MAPK1, NOD2, HSF1, JUN, MAPK14, NKX2-1, IRF3, SCARB1
GO:0001892~embryonic placenta development	8	1.47E-04	0.002490691	GATA2, HIF1A, CEBPB, HSF1, EPAS1, SP1, SP3, PRDM1
GO:0051170~nuclear import	15	1.47E-04	0.002499829	SIX3, TP53, PML, SIX2, ARNTL, TGFB1, MAPK1, IPO7, JUN, TRPS1, IPO5, BCL3, BCL6, RERE, TOB1
GO:0045792~negative regulation of cell size	16	1.52E-04	0.002553153	NRP1, PPARG, TP53, SMAD4, PML, SMAD3, TP63, NPR1, RB1, TGFB1, CDKN1A, TSC1, SEMA4F, BCL2, BCL6, SEMA3A
GO:0051153~regulation of striated muscle cell differentiation	9	1.63E-04	0.002682306	HDAC5, HDAC4, HDAC3, HDAC1, BCL2, EZH2, EDN1, HDAC9, PROX1
GO:0030335~positive regulation of cell migration	15	1.66E-04	0.002719466	ADAM10, EDN1, SMAD3, TAC1, IL6R, TGFB1, IGF1R, MAPK1, MIA3, HIF1A, BCL2, VEGFA, SCARB1, HDAC9, HDAC6
GO:0048872~homeostasis of number of cells	16	1.70E-04	0.002767471	LIPA, LYN, EPAS1, STAT5A, RB1, BCL2L11, TGFB1, BAK1, TAL1, SP1, SP3, BCL2, VEGFA, BCL6, FAS, FH
GO:0070302~regulation of stress-activated protein kinase signaling pathway	13	1.82E-04	0.002941689	HDAC3, NOD2, ERCC6, LYN, HIPK3, HIPK2, EDN1, MAP3K10, SEMA4C, MAPK8IP3, ZEB2, NCOR1, PDCD4

GO:0019216~regulation of lipid metabolic process	17	1.87E-04	0.0030023	PPARA, STAT5A, PRKAG2, PPARG, SF1, NFKB1, RB1, ABCG4, PROX1, ABCG1, TGFB1, BRCA1, NR1H2, CDC42, RAC1, NR1H3, NR5A1
GO:0002761~regulation of myeloid leukocyte differentiation	10	1.88E-04	0.003006953	ZBTB7A, ESRRA, JUN, STAT5A, MITF, KITLG, RB1, RUNX1, TOB2, CTNNB1
GO:0016202~regulation of striated muscle tissue development	11	1.90E-04	0.003022819	HDAC5, HDAC4, HDAC3, HDAC1, TBX5, BCL2, GATA4, SMAD3, HDAC9, TGFB1, TWIST1
GO:0010743~regulation of foam cell differentiation	8	1.95E-04	0.003089962	NR1H2, PPARA, ITGAV, PPARG, NFKB1, ITGB3, ABCG1, NR1H3
GO:0042770~DNA damage response, signal transduction	14	2.02E-04	0.003182815	TP53, PML, TP63, CHEK1, MBD4, ATM, BRCA1, ERCC6, XPC, NUPR1, MAPK14, HIPK2, BCL3, ABL1
GO:0001709~cell fate determination	9	2.06E-04	0.003225526	CDC42, GATA2, MCL1, PAX6, DSCAML1, PAX2, PROX1, KLF4, CTNNB1
GO:0001932~regulation of protein amino acid phosphorylation	22	2.16E-04	0.003371806	LYN, SMAD7, PRKAG2, EDN1, SMAD4, KITLG, ZEB2, IL6R, RICTOR, PDCD4, TGFB1, BAK1, PRKAR2A, NOD2, ERCC6, CCND2, JUN, HIPK3, BCL2, MAP3K10, MAPK8IP3, BMPR1A
GO:0043392~negative regulation of DNA binding	11	2.26E-04	0.003506503	HDAC4, KDM1A, NOD2, THRA, TNFSF4, HDAC2, SMAD7, PBX1, NR0B1, PROX1, SIRT1
GO:0048634~regulation of muscle development	11	2.26E-04	0.003506503	HDAC5, HDAC4, HDAC3, HDAC1, TBX5, BCL2, GATA4, SMAD3, HDAC9, TGFB1, TWIST1
GO:0032844~regulation of homeostatic process	17	2.31E-04	0.003530145	LYN, MAFB, SMAD7, STAT5A, SPI1, TAC1, CDK6, FOXO3, TGFB1, BAK1, TAL1, HIF1A, ETS1, MAPK14, BCL2, VEGFA, MYC
GO:0046649~lymphocyte activation	24	2.30E-04	0.003540043	EGR1, KLF6, TNFSF4, STAT5A, TP53, SMAD3, SOX4, TGFB1, CTNNB1, HDAC5, HDAC4, BAK1, CHD7, SP3, BCL2, BCL11B, IRF1, BCL3, PATZ1, BCL6, FAS, IRF4, HDAC9, TCF3
GO:0030308~negative regulation of cell growth	15	2.38E-04	0.00361322	NRP1, PPARG, TP53, SMAD4, PML, SMAD3, TP63, NPR1, RB1, TGFB1, CDKN1A, SEMA4F, BCL2, BCL6, SEMA3A
GO:0007178~transmembrane receptor protein serine/threonine kinase signaling pathway	16	2.38E-04	0.00362174	SMAD7, CREB1, SMAD4, PML, SMAD3, SKI, SMAD2, TGFB1, FOS, JUN, HIPK2, COL1A2, SMURF2, SMURF1, BMPR1A, TOB1
GO:0080135~regulation of cellular response to stress	16	2.38E-04	0.00362174	LYN, EDN1, ZEB2, PDCD4, SIRT1, BRCA1, NTRK3, HDAC3, NOD2, ERCC6, HIPK3, HIPK2, SEMA4C, MAP3K10, MAPK8IP3, NCOR1
GO:0000060~protein import into nucleus, translocation	9	2.57E-04	0.003885139	MAPK1, JUN, PML, TP53, BCL3, BCL6, ARNTL, TGFB1, TOB1
GO:0030099~myeloid cell differentiation	15	2.67E-04	0.004019939	EPAS1, LYN, PPARG, PML, RB1, TGFB1, TAL1, CDC42, SP1, SP3, VEGFA, BCL6, IRF4, TRAF6, RUNX1
GO:0048536~spleen development	7	2.74E-04	0.004107927	BCL2, NKX3-2, BCL3, PBX1, NFKB2, FAS, BCL2L11
GO:0070482~response to oxygen levels	19	3.22E-04	0.004798186	PPARA, SLC8A1, EPAS1, VHL, EDN1, CREBBP, NR4A2, SMAD4, PML, SMAD3, USF1, TGFB1, CDKN1A, EP300, HIF1A, TFRC, BCL2, VEGFA, CAMK2D
GO:0030879~mammary gland development	12	3.24E-04	0.004812507	PGR, IGF1R, AGPAT6, IRF6, ERBB3, ERBB2, CREB1, STAT5A, HK2, USF2, BCL2L11, TGFB1
GO:0048168~regulation of neuronal synaptic plasticity	9	3.91E-04	0.00577802	EGR1, ARC, EGR2, GRIN2B, NCDN, RAB11A, BHLHE40, DBN1, EPHB2
GO:0002237~response to molecule of bacterial origin	14	4.21E-04	0.006130539	TG, RELA, TAC1, IL6R, STAT1, FOS, MAPK1, NOD2, HSF1, JUN, MAPK14, NKX2-1, IRF3, SCARB1
GO:0043408~regulation of MAPKKK cascade	16	4.44E-04	0.006445467	ERBB2, EDN1, ZEB2, IL6R, PDCD4, CTNNB1, NOD2, HDAC3, ERCC6, GRIN2B, HIPK3, HIPK2, SEMA4C, MAP3K10, MAPK8IP3, NCOR1
GO:0070201~regulation of establishment of protein localization	17	4.58E-04	0.006616237	TNFSF4, SMAD4, SMAD3, RHOQ, CDH1, MXI1, APPL1, SIRT1, UHMK1, TGFB1, NOD2, WWP2, RHOA, RAB11A, BCL3, TRAF6, BMPR1A
GO:0007519~skeletal muscle tissue development	12	4.92E-04	0.007043804	APP, FOXL2, EP300, ERBB3, DNER, MAPK14, ERBB2, TAZ, SIX1, RHOA, NR2F2, FOXP2

GO:0060538~skeletal muscle organ development	12	4.92E-04	0.007043804	APP, FOXL2, EP300, ERBB3, DNER, MAPK14, ERBB2, TAZ, SIX1, RHOA, NR2F2, FOXP2
GO:0007568~aging	16	4.91E-04	0.007054696	NOX4, RELA, TP53, PML, TP63, SRF, SIRT1, PTEN, PDCD4, TGFB1, SREBF2, FOS, TFRC, ZMIZ1, JUN, BCL2
GO:0001666~response to hypoxia	18	5.03E-04	0.00711204	PPARA, SLC8A1, EPAS1, VHL, EDN1, CREBBP, NR4A2, SMAD4, PML, SMAD3, USF1, TGFB1, EP300, HIF1A, TFRC, BCL2, VEGFA, CAMK2D
GO:0009612~response to mechanical stimulus	11	4.99E-04	0.007117656	FOS, GRIN2B, BTG2, RELA, JUN, MAP1B, COL1A1, STAT1, FOSL1, MYC, FOXP2
GO:0051318~G1 phase	7	5.02E-04	0.007133267	E2F1, CDKN1C, TAF1, MDM2, CDK6, RB1, TCF3
GO:0034983~peptidyl-lysine deacetylation	4	5.19E-04	0.007312201	HDAC4, HDAC9, SIRT1, HDAC6
GO:0031344~regulation of cell projection organization	14	5.90E-04	0.008269552	NTRK3, CDC42, NRP1, YWHAH, SEMA4F, MAP1B, RAC1, RHOA, RHOQ, SEMA3A, SEMA4D, CDH4, DBN1, EPHB2
GO:0006730~one-carbon metabolic process	16	5.96E-04	0.008313297	PPME1, MAT2A, RAB3D, EZH2, CTCF, SUZ12, ATRX, KDM1A, FOS, DHFR, BTG2, DNMT1, KDM4A, RNF20, BAZ2A, SUV420H1
GO:0002009~morphogenesis of an epithelium	15	6.33E-04	0.008762058	TBX5, SMAD4, TP63, ZEB2, PAX2, PROX1, CTNNB1, TSC1, BCL2, SIX1, TGIF1, TFAP2A, PBX1, TRAF6, TWIST1
GO:0030155~regulation of cell adhesion	18	6.51E-04	0.008967547	ADAM10, CYTH1, ERBB3, SMAD7, STAT5A, ERBB2, CCDC80, SMAD3, CDK6, PTEN, TGFB1, MIA3, TSC1, BCL2, BCL6, COL1A1, RASA1, ALOX12
GO:0030855~epithelial cell differentiation	18	6.51E-04	0.008967547	E2F4, FOXA2, PPARG, FOXA1, PAX6, TP63, PAX2, PROX1, WT1, PGR, AGPAT6, IRF6, JUN, VEGFA, SIX1, MSI1, NKX2-1, NR2F2
GO:0048538~thymus development	7	6.61E-04	0.009036017	CRKL, BCL11B, BCL2, SIX1, PBX1, BCL2L11, CTNNB1
GO:0045930~negative regulation of mitotic cell cycle	7	6.61E-04	0.009036017	BCL2, PML, SMAD3, BCL6, FOXC1, RB1, TGFB1
GO:0045768~positive regulation of anti-apoptosis	8	6.61E-04	0.009071468	CDKN1A, BTG2, SMAD7, LIFR, IL6R, SIRT1, RASA1, SLC9A1
GO:0042771~DNA damage response, signal transduction by p53 class mediator resulting in induction of apoptosis	6	6.88E-04	0.009366894	NUPR1, HIPK2, PML, TP53, TP63, BCL3
GO:0017015~regulation of transforming growth factor beta receptor signaling pathway	9	6.94E-04	0.009412403	CDKN1C, HIPK2, SMAD4, SMAD3, SKI, SMAD2, SMURF2, ZEB1, TGFB1
GO:0010717~regulation of epithelial to mesenchymal transition	5	7.07E-04	0.009546404	SMAD7, SMAD4, SMAD3, SMAD2, TGFB1
GO:0042110~T cell activation	17	7.21E-04	0.009688553	EGR1, TNFSF4, STAT5A, TP53, SMAD3, SOX4, TGFB1, CTNNB1, CHD7, SP3, BCL2, BCL11B, IRF1, BCL3, PATZ1, FAS, IRF4
GO:0045927~positive regulation of growth	13	7.45E-04	0.009930731	ADAM10, STAT5A, CREB1, TAF9B, MAP1B, PROX1, CDH4, NTRK3, EP300, CHD7, HSF1, BCL2, ALOX12
GO:0033554~cellular response to stress	48	7.62E-04	0.010122519	HMGB1, PML, TP63, CHEK1, FOXO3, PTTG1, TTC5, KIN, FOS, ERCC6, RAD21, BCL2, GPX3, BCL3, AATF, BCL6, SREBF1, POLK, EPAS1, LYN, MAP1B, MAP2K4, PAPD7, TP53, MBD4, SIRT7, SIRT1, BRCA1, SMC3, ATM, ATRX, MAPK1, CDKN1A, CRKL, HIF1A, XPC, HDAC2, NUPR1, BTG2, MAPK14, JUN, HIPK2, MAP3K10, MAPK8IP3, ABL1, PARP1, HDAC6, REV3L
GO:0051100~negative regulation of binding	11	7.67E-04	0.010146643	HDAC4, KDM1A, NOD2, THRA, TNFSF4, HDAC2, SMAD7, PBX1, NR0B1, PROX1, SIRT1
GO:0048545~response to steroid hormone stimulus	22	8.80E-04	0.011543988	ERBB2, RELA, PPARG, MAP1B, ESR1, IL6R, AQP1, PTEN, BRCA1, STAT3, TGFB1, CTNNB1, MAPK1, FOS, CDKN1A, EP300, CCND2, GATA3, BCL2, FAS, COL1A1, FOSL1
GO:0007612~learning	11	8.79E-04	0.011573293	ATXN1, FOS, APP, GRIN2B, JUN, ATP1A3, TAC1, FOSL1, EPHB2, FOXP2, VDAC1
GO:0009953~dorsal/ventral pattern formation	11	8.79E-04	0.011573293	FOXA2, LHX2, EDN1, PAX6, TGIF1, NKX2-1, DSCAML1, SMAD2, GLI1, CTNNB1, BMPR1A
GO:0050678~regulation of epithelial cell proliferation	12	9.29E-04	0.01214277	CDKN1C, PGR, CCND2, ERBB2, VEGFA, PAX6, SMAD3, CDK6, RUNX3, TGFB1, BMPR1A, FOXP2

GO:0016337~cell-cell adhesion	28	0.00101	0.013119475	PCDHA6, PCDHA7, PCDHA8, CLDN18, THRA, PCDHA9, PCDHA2, PCDHA3, PCDHA4, PCDHA5, DSCAML1, CDH1, PCDHA1, SOX9, CDH4, PCDHAC2, CTNNB1, PCDHAC1, CDH20, PVRL1, BCL2, PCDHA10, PCDHA11, PCDHA12, PCDHA13, PCDH11Y, PCDH11X, PCDH10, SIGLEC1, CD34, ITGA5, DSC2, NCAN
GO:0010627~regulation of protein kinase cascade	26	0.00107	0.01377643	ERBB3, ERBB2, EDN1, ZEB2, PTEN, PDCD4, TGFB1, CTNNB1, NOD2, ERCC6, GRIN2B, MIER1, RHOA, TRAF6, LYN, RELA, IL6R, RICTOR, HDAC3, HIPK3, HIPK2, SEMA4C, MAP3K10, MAPK8IP3, TNFAIP3, NCOR1
GO:0045666~positive regulation of neuron differentiation	7	0.00109	0.014041772	PHOX2B, FOXA2, HOXD3, FOXA1, MAP1B, RHOA, TGIF1
GO:0017038~protein import	17	0.0011	0.014109309	YWHAZ, SIX3, TP53, PML, SIX2, ARNTL, TGFB1, MAPK1, IPO7, TOMM70A, JUN, TRPS1, IPO5, BCL3, BCL6, RERE, TOB1
GO:0022415~viral reproductive process	11	0.00114	0.014573184	CTBP2, PVRL1, WWP2, SMARCB1, HIPK2, SMAD3, HCFC1, USF1, HMGA1, USF2, TGFB1
GO:0051591~response to cAMP	9	0.00116	0.01477409	FOS, MAT2A, CCND2, RELA, JUN, IL6R, COL1A1, STAT1, FOSL1
GO:0007569~cell aging	8	0.00121	0.015350628	NOX4, ZMIZ1, BCL2, PML, TP53, TP63, PDCD4, SIRT1
GO:0014033~neural crest cell differentiation	8	0.00121	0.015350628	HIF1A, HAND2, EDN1, CYP26A1, KITLG, ZEB2, FOXC1, SOX9
GO:0014032~neural crest cell development	8	0.00121	0.015350628	HIF1A, HAND2, EDN1, CYP26A1, KITLG, ZEB2, FOXC1, SOX9
GO:0031667~response to nutrient levels	22	0.00122	0.01542134	SREBF1, PPARA, CYP24A1, SLC8A1, RELA, PPARG, MAP1B, TP53, STAT1, USF1, SIRT1, PTEN, USF2, AQP3, TGFB1, VDR, TFRC, JUN, BCL2, COL1A1, KLF4, HDAC6
GO:0046907~intracellular transport	53	0.00124	0.015596273	CHMP7, EIF5A, TGFB1, TAPBP, HOOK3, BAK1, APP, SRPR, RAE1, GOLGA3, STX6, SIX3, TP53, SIX2, ARNTL, STXBP4, VTI1A, ERGIC2, MAPK1, KIF1B, CAMK4, IPO7, JUN, IPO5, ZNF384, RAB14, CUX1, YWHAZ, PML, KLC2, SEC63, AP3M1, BCL2, PIKFYVE, SEC22A, BCL3, RAB11A, BCL6, SMG7, ABCG1, ATXN1, YWHAH, ARF1, TSC1, ATP2A2, TOMM70A, RAB35, TRPS1, SMURF1, GGA2, RERE, HDAC6, TOB1
GO:0010871~negative regulation of receptor biosynthetic process	4	0.00125	0.015625521	PPARA, ITGAV, PPARG, ITGB3
GO:0010832~negative regulation of myotube differentiation	4	0.00125	0.015625521	HDAC5, HDAC4, HDAC3, HDAC1
GO:0010830~regulation of myotube differentiation	4	0.00125	0.015625521	HDAC5, HDAC4, HDAC3, HDAC1
GO:0007517~muscle organ development	23	0.00127	0.015776164	FOXL2, ERBB3, SMAD7, TBX5, ERBB2, TAZ, TP63, SIRT1, PTEN, PROX1, FOXP2, FXR1, APP, EP300, TSC1, MAPK14, DNER, TEAD4, SIX1, RHOA, FOXC1, NR2F2, TCF12
GO:0010594~regulation of endothelial cell migration	7	0.00138	0.017009746	HDAC5, DLL4, VEGFA, EDN1, SCARB1, HDAC9, TGFB1
GO:0043331~response to dsRNA	7	0.00138	0.017009746	MAPK1, NOD2, SMAD3, IRF3, SMAD2, LIN28A, STAT1
GO:0033157~regulation of intracellular protein transport	10	0.00137	0.017049343	RHOA, SMAD4, SMAD3, BCL3, CDH1, MXI1, SIRT1, UHMK1, TGFB1, BMPR1A
GO:0045926~negative regulation of growth	15	0.00148	0.018066683	NRP1, PPARG, TP53, SMAD4, PML, SMAD3, TP63, NPR1, RB1, TGFB1, CDKN1A, SEMA4F, BCL2, BCL6, SEMA3A
GO:0008585~female gonad development	11	0.00147	0.01807606	PGR, FOXL2, CCND2, BCL2, STAT5A, VEGFA, FOXC1, FOXO3, SIRT1, LFNG, NR5A1
GO:0048167~regulation of synaptic plasticity	11	0.00147	0.01807606	EGR1, ARC, YWHAH, EGR2, GRIN2B, NCDN, MAP1B, RAB11A, BHLHE40, DBN1, EPHB2
GO:0009314~response to radiation	22	0.00148	0.018093475	MAT2A, RELA, TP53, ATP1A3, PML, MBD4, IL6R, USF1, BRCA1, TGFB1, ATM, ATXN1, BAK1, FOS, CDKN1A, APP, ERCC6, XPC, BCL2, BCL3, MYC, CLOCK
GO:0000302~response to reactive oxygen species	12	0.00148	0.018114296	FOS, SLC8A1, ERCC6, EP300, RELA, JUN, BCL2, GPX3, COL1A1, STAT1, FOSL1, HDAC6
GO:0001525~angiogenesis	18	0.00155	0.018827822	KLF5, GNA13, NRP1, EPAS1, VHL, EDN1, ENPEP, SRF, PTEN, CTNNB1, HDAC3, HIF1A, HAND2, MAPK14, DLL4, JUN, VEGFA, RBPJ
GO:0042476~odontogenesis	10	0.00157	0.019036102	ERBB3, COL1A2, TP63, FOXC1, COL1A1, TRAF6, BCL2L11, TGFB1, CTNNB1, BMPR1A

GO:0042306~regulation of protein import into nucleus	9	0.0016	0.019210059	RHOA, SMAD4, SMAD3, BCL3, CDH1, MXI1, SIRT1, TGFB1, BMPR1A
GO:0031669~cellular response to nutrient levels	9	0.0016	0.019210059	SREBF1, VDR, CYP24A1, JUN, TP53, USF1, SIRT1, USF2, HDAC6
GO:0006605~protein targeting	23	0.00161	0.019359403	YWHAZ, SIX3, TP53, PML, EIF5A, SIX2, STXBP4, ARNTL, TGFB1, SEC63, MAPK1, SRPR, TOMM70A, IPO7, AP3M1, JUN, TRPS1, IPO5, BCL3, BCL6, SMURF1, RERE, TOB1
GO:0007611~learning or memory	15	0.00162	0.01936076	EGR1, EGR2, ATP1A3, TAC1, PTEN, VDAC1, EPHB2, FOXP2, ATXN1, FOS, APP, GRIN2B, ITGA5, JUN, FOSL1
GO:0051493~regulation of cytoskeleton organization	17	0.00164	0.019554115	EDN1, MAP1B, SMAD3, TAC1, RHOQ, RICTOR, CAPZB, PROX1, BRCA1, CTNNB1, MYCBP2, TSC1, RAC1, RHOA, RASA1, ADD2, HDAC6
GO:0046328~regulation of JNK cascade	11	0.00166	0.019666001	HDAC3, NOD2, ERCC6, HIPK3, HIPK2, EDN1, MAP3K10, MAPK8IP3, ZEB2, PDCD4, NCOR1
GO:0045648~positive regulation of erythrocyte differentiation	5	0.00171	0.020164795	TAL1, HIF1A, ETS1, MAPK14, FOXO3
GO:0010559~regulation of glycoprotein biosynthetic process	5	0.00171	0.020164795	BCL2, AATF, NECAB3, TCF7L2, CTNNB1
GO:0030330~DNA damage response, signal transduction by p53 class mediator	7	0.00171	0.020170209	NUPR1, HIPK2, PML, TP53, TP63, BCL3, BRCA1
GO:0001837~epithelial to mesenchymal transition	6	0.00173	0.020278048	HIF1A, TRIM28, SOX9, TGFB1, CTNNB1, NFATC1
GO:0006342~chromatin silencing	6	0.00173	0.020278048	HDAC5, HDAC2, SIRT6, SIRT7, BAZ2A, SIRT1
GO:0000080~G1 phase of mitotic cell cycle	6	0.00173	0.020278048	E2F1, CDKN1C, TAF1, MDM2, CDK6, TCF3
GO:0007492~endoderm development	6	0.00173	0.020278048	ARC, PAX9, NKX2-1, TGFB1, CTNNB1, BMPR1A
GO:0048145~regulation of fibroblast proliferation	8	0.00175	0.020436137	BMI1, CDKN1A, FOSL2, NUPR1, ZMIZ1, TP53, TGIF1, CDK6
GO:0031016~pancreas development	8	0.00175	0.020436137	FOXA2, NKX3-2, SOX4, SMAD2, IL6R, PROX1, TCF7L2, CTNNB1
GO:0048705~skeletal system morphogenesis	15	0.00177	0.020584605	THRA, SIX2, DSCAML1, ZEB1, SOX9, TGFB1, HOXC9, HOXD3, SIX1, NKX3-2, TFAP2A, COL1A1, RUNX2, MYC, PLEKHA1
GO:0048706~embryonic skeletal system development	12	0.00184	0.021321121	HOXC9, SP1, SP3, HOXD3, SIX1, NKX3-2, DSCAML1, TFAP2A, SIX2, PBX1, ZEB1, COL1A1
GO:0046890~regulation of lipid biosynthetic process	9	0.00185	0.021462768	NR1H2, SF1, NFKB1, PROX1, ABCG4, ABCG1, BRCA1, NR5A1, NR1H3
GO:0050768~negative regulation of neurogenesis	9	0.00185	0.021462768	NRP1, YWHAH, SEMA4F, RHOA, TP53, SEMA3A, TGFB1, EPHB2, BMPR1A
GO:0051099~positive regulation of binding	12	0.00204	0.023431299	HDAC5, HDAC4, NOD2, EP300, MTDH, SMARCB1, RELA, HIPK2, SKI, IRF4, ADD2, TGFB1
GO:0042542~response to hydrogen peroxide	10	0.00205	0.023438805	SLC8A1, EP300, RELA, JUN, BCL2, GPX3, COL1A1, STAT1, FOSL1, HDAC6
GO:0000082~G1/S transition of mitotic cell cycle	10	0.00205	0.023438805	BCAT1, CDKN1A, E2F4, CCND2, E2F6, BCL2, CAMK2D, RB1, LATS2, NFATC1
GO:0060562~epithelial tube morphogenesis	11	0.00209	0.023792604	TSC1, BCL2, SIX1, SMAD4, TGIF1, TFAP2A, ZEB2, PBX1, TRAF6, PAX2, TWIST1
GO:0051223~regulation of protein transport	15	0.00209	0.023845797	TNFSF4, SMAD4, SMAD3, CDH1, MXI1, SIRT1, UHMK1, TGFB1, NOD2, WWP2, RHOA, RAB11A, BCL3, TRAF6, BMPR1A
GO:0001952~regulation of cell-matrix adhesion	7	0.00211	0.023859858	TSC1, BCL2, SMAD3, CDK6, BCL6, PTEN, RASA1

GO:0043254~regulation of protein complex assembly	13	0.00212	0.023955859	TAF1, HMGB1, MAP1B, HCFC1, RICTOR, SRF, CAPZB, CDC42, EIF4EBP1, RAC1, RASA1, ADD2, HDAC6
GO:0045834~positive regulation of lipid metabolic process	9	0.00215	0.024138419	NR1H2, CDC42, PPARA, PPARG, RAC1, ABCG4, ABCG1, TGFB1, NR1H3
GO:0010810~regulation of cell-substrate adhesion	9	0.00215	0.024138419	TSC1, BCL2, CCDC80, SMAD3, CDK6, BCL6, COL1A1, PTEN, RASA1
GO:0048609~reproductive process in a multicellular organism	41	0.00224	0.025135432	STAT5A, ERBB2, HK2, TAC1, PAX5, CHEK1, TBP, FOXO3, PTTG1, TGFB1, WT1, GLI1, PGR, AGPAT6, HSF1, HSF2, BCL2, SPATA2, HOXA10, NKX2-1, BCL6, LFNG, FOXL2, CREB1, AFF4, SIX5, NR0B1, SIRT1, ATM, USF2, BCL2L11, SREBF2, GMCL1, SP1, CCND2, ZMIZ1, SP3, VEGFA, FOXC1, TOB2, NR5A1
GO:0060173~limb development	14	0.00233	0.026002702	TBX5, TP63, SKI, GJA5, CTNNB1, BAK1, CHD7, FBXW4, HOXA10, PBX1, NR2F2, PBX2, TWIST1, BMPR1A
GO:0048736~appendage development	14	0.00233	0.026002702	TBX5, TP63, SKI, GJA5, CTNNB1, BAK1, CHD7, FBXW4, HOXA10, PBX1, NR2F2, PBX2, TWIST1, BMPR1A
GO:0007050~cell cycle arrest	14	0.00233	0.026002702	CDKN1C, CDKN1A, KAT2B, IRF6, PML, TP53, SMAD3, RB1, MYC, TCF7L2, TGFB1, UHMK1, TP53INP1, SESN3
GO:0007049~cell cycle	59	0.00244	0.027103202	E2F1, CCNT2, E2F2, E2F3, E2F4, E2F6, RBM7, PTTG1, SENP5, LATS2, TGFB1, CTNNB1, APP, RAD21, MYC, SF1, PAPD7, TP53, BANP, CDK6, RB1, MAPK1, EP300, CCND2, MDM2, BCAT1, FOXM1, PML, HCFC1, CHEK1, TCF7L2, UHMK1, SESN3, SMARCB1, BCL2, CAMK2D, THAP1, LFNG, TCF3, NFATC1, TAF1, PDS5B, KAT2B, CKAP5, SMAD3, SIRT7, APPL1, TET2, SMC3, BRCA1, ATM, CDKN1C, HDAC3, CDKN1A, IRF6, RNF2, ABL1, NCOR1, TP53INP1
GO:0048048~embryonic eye morphogenesis	5	0.00245	0.02720838	FOXL2, SP1, SP3, ZEB1, PROX1
GO:0048638~regulation of developmental growth	9	0.00247	0.027308266	NTRK3, NRP1, SEMA4F, TBX5, GATA4, MAP1B, SEMA3A, PROX1, CDH4
GO:0043414~biopolymer methylation	11	0.00262	0.028809568	ATRX, SUZ12, FOS, RAB3D, BTG2, EZH2, DNMT1, CTCF, BAZ2A, RNF20, SUV420H1
GO:0046822~regulation of nucleocytoplasmic transport	10	0.00263	0.028820861	RHOA, SMAD4, SMAD3, BCL3, CDH1, MXI1, SIRT1, UHMK1, TGFB1, BMPR1A
GO:0030518~steroid hormone receptor signaling pathway	10	0.00263	0.028820861	PGR, AR, YWHAH, RNF4, TAF7, ESR1, FHL2, RB1, BRCA1, CTNNB1
GO:0035282~segmentation	9	0.00284	0.030966171	KAT2A, BMI1, EP300, EGR2, MAFB, ZEB2, RBPJ, ATM, MLLT3
GO:0030516~regulation of axon extension	6	0.00287	0.031030325	NTRK3, NRP1, SEMA4F, MAP1B, SEMA3A, CDH4
GO:0001825~blastocyst formation	6	0.00287	0.031030325	TJP1, SP1, SP3, TEAD4, CDH1, SRF
GO:0010608~posttranscriptional regulation of gene expression	22	0.00285	0.031037351	SMAD7, VHL, PML, SMAD3, EIF5A, SOX4, ELAVL1, SMAD2, CPEB1, IGF2BP3, LIN28A, PTEN, YBX1, EIF4B, MAPK1, EIF4EBP1, APP, EIF4EBP2, TSC1, BCL2, VEGFA, BCL3
GO:0045767~regulation of anti-apoptosis	8	0.00287	0.031118593	CDKN1A, BTG2, SMAD7, LIFR, IL6R, SIRT1, RASA1, SLC9A1
GO:0010975~regulation of neuron projection development	11	0.00292	0.031443071	NTRK3, NRP1, YWHAH, SEMA4F, MAP1B, RHOA, SEMA3A, SEMA4D, CDH4, DBN1, EPHB2
GO:0043388~positive regulation of DNA binding	11	0.00292	0.031443071	HDAC5, HDAC4, NOD2, EP300, MTDH, SMARCB1, RELA, HIPK2, SKI, IRF4, TGFB1
GO:0007369~gastrulation	11	0.00292	0.031443071	FOXA2, RNF2, GATA4, SMAD4, TP53, SMAD3, SMAD2, FOXC1, KLF4, CTNNB1, BMPR1A
GO:0050769~positive regulation of neurogenesis	10	0.00296	0.031824022	NTRK3, LYN, RELA, PPARG, MAP1B, RHOA, PAX6, SEMA4D, CDH4, EPHB2
GO:0007398~ectoderm development	21	0.00317	0.033829361	PPARA, FOXA2, RELA, KLK5, PAX6, TP63, SOX9, GLI1, CTNNB1, ATP2A2, IRF6, CASP14, BCL2, BNC1, COL1A2, TFAP2A, COL1A1, SMURF1, KLF4, RUNX3, BMPR1A
GO:0016032~viral reproduction	11	0.00324	0.034462341	CTBP2, PVRL1, WWP2, SMARCB1, HIPK2, SMAD3, HCFC1, USF1, HMGA1, USF2, TGFB1
GO:0001890~placenta development	10	0.00333	0.035264151	VDR, GATA2, HIF1A, CEBPB, HSF1, EPAS1, SP1, SP3, PPARG, PRDM1

GO:0007179~transforming growth factor beta receptor signaling pathway	10	0.00333	0.035264151	FOS, JUN, CREB1, COL1A2, PML, SMAD4, SMAD3, SMAD2, TGFB1, BMPR1A
GO:0043583~ear development	13	0.00336	0.035415547	MAFB, EDN1, SIX2, PAX2, PROX1, BCL2L11, TGFB1, CHD7, BCL2, SIX1, NKX3-2, CUX1, MYC
GO:0010595~positive regulation of endothelial cell migration	5	0.0034	0.035791579	VEGFA, EDN1, SCARB1, HDAC9, TGFB1
GO:0009611~response to wounding	43	0.00343	0.036006624	GNA13, PPARA, YWHAZ, NRP1, FOXA2, ERBB3, ERBB2, CXCL9, TAC1, AFAP1L2, NFKB1, ITGB3, SGMS1, SRF, TGFB1, CCL26, FOS, MIA3, BCL2, RAC1, SCARB1, NFATC3, NOX4, CIITA, KLF6, CEBPB, TNFSF4, LIPA, LYN, RELA, MAP1B, SMAD3, IL6R, STAT3, HDAC5, HDAC4, SIGLEC1, CCR7, HIF1A, NUPR1, TFRC, ITGA5, HDAC9
GO:0001836~release of cytochrome c from mitochondria	6	0.00361	0.037690167	BAK1, JUN, BCL2, TP53, SMAD3, MYC
GO:0002064~epithelial cell development	6	0.00361	0.037690167	PGR, AGPAT6, E2F4, FOXA1, TP63, PAX2
GO:0045814~negative regulation of gene expression, epigenetic	6	0.00361	0.037690167	HDAC5, HDAC2, SIRT6, SIRT7, BAZ2A, SIRT1
GO:0051174~regulation of phosphorus metabolic process	40	0.00368	0.038312288	CCNT2, ERBB2, EDN1, PRKAG2, KITLG, CHEK1, ZEB2, PDCD4, PTEN, LATS2, TGFB1, CDC42, BAK1, PRKAR2A, APP, NOD2, ERCC6, BCL2, RAC1, TRAF6, LYN, SMAD7, TAF7, SMG7, SMAD4, SMAD3, RB1, IL6R, RICTOR, ATXN1, CDKN1C, CDKN1A, TSC1, CCND2, RGS3, JUN, HIPK3, MAP3K10, MAPK8IP3, BMPR1A
GO:0051341~regulation of oxidoreductase activity	8	0.00388	0.040070106	VDR, HIF1A, EDN1, NFKB1, SCARB1, ABL1, ABL2, HDAC6
GO:0046661~male sex differentiation	11	0.00398	0.041006088	AR, BCL2, STAT5A, SF1, NKX2-1, SOX9, NR0B1, WT1, BCL2L11, BMPR1A, NR5A1
GO:0010770~positive regulation of cell morphogenesis involved in differentiation	4	0.00404	0.041514409	SMAD4, SMAD3, SMAD2, TGFB1
GO:0014065~phosphoinositide 3-kinase cascade	4	0.00404	0.041514409	IGF1R, ERBB3, ERBB2, EDN1
GO:0010718~positive regulation of epithelial to mesenchymal transition	4	0.00404	0.041514409	SMAD4, SMAD3, SMAD2, TGFB1
GO:0001655~urogenital system development	14	0.00416	0.042539067	AR, FOXA1, SMAD4, SIX2, TP63, PAX2, PROX1, BCL2L11, WT1, TSC1, BCL2, SIX1, PBX1, FOXC1
GO:0060348~bone development	15	0.00423	0.043113565	CYP24A1, THRA, FHL2, SMAD3, SOX9, TGFB1, GLI1, SP1, SP3, BCL2, JUND, FOXC1, COL1A1, TRAF6, RUNX2
GO:0010035~response to inorganic substance	21	0.00445	0.045143195	SLC8A1, RELA, MAP1B, NR4A2, STIM1, STAT1, PTEN, AQP3, CTNNB1, SREBF2, FOS, CDKN1A, ERCC6, EP300, TFRC, JUN, BCL2, GPX3, COL1A1, FOSL1, HDAC6
GO:0045668~negative regulation of osteoblast differentiation	5	0.00457	0.046118664	SMAD3, CDK6, SKI, TWIST2, TOB1
GO:0032330~regulation of chondrocyte differentiation	5	0.00457	0.046118664	MAF, RELA, SOX9, RUNX2, CTNNB1
GO:0045765~regulation of angiogenesis	10	0.00465	0.046774224	HDAC5, WARS, GATA2, HIF1A, ERBB2, GATA4, PML, NPR1, HDAC9, RUNX1
GO:0008584~male gonad development	9	0.00472	0.047193365	AR, BCL2, SF1, NKX2-1, SOX9, NR0B1, WT1, BCL2L11, NR5A1
GO:0035107~appendage morphogenesis	13	0.00471	0.047226558	TBX5, TP63, SKI, GJA5, CTNNB1, BAK1, CHD7, FBXW4, HOXA10, PBX1, PBX2, TWIST1, BMPR1A
GO:0035108~limb morphogenesis	13	0.00471	0.047226558	TBX5, TP63, SKI, GJA5, CTNNB1, BAK1, CHD7, FBXW4, HOXA10, PBX1, PBX2, TWIST1, BMPR1A

Supplementary Table S3

Pathway	Count	PValue	Benjamini	Genes
hsa04722:Neurotrophin signaling pathway	23	3.06E-07	6.08E-06	YWHAZ, RELA, TP53, RAF1, NFKB1, FOXO3, NTRK3, CDC42, MAPK1, CRKL, YWHAH, CAMK4, MAPK14, JUN, BCL2, RAC1, RHOA, CAMK2D, RAP1A, TRAF6, ABL1, PIK3R3, AKT3
hsa04012:ErbB signaling pathway	19	3.56E-07	6.19E-06	ERBB3, ERBB2, STAT5A, MAP2K4, RAF1, ELK1, PAK6, MAPK1, PAK7, CDKN1A, EIF4EBP1, CRKL, JUN, CAMK2D, ABL1, PIK3R3, ABL2, MYC, AKT3
hsa04520:Adherens junction	16	7.63E-06	7.57E-05	ERBB2, CREBBP, SMAD4, SMAD3, CDH1, SMAD2, TCF7L2, CTNNB1, CDC42, IGF1R, MAPK1, TJP1, EP300, PVRL1, RAC1, RHOA
hsa04360:Axon guidance	20	3.33E-05	2.72E-04	PLXNC1, NRP1, GNAI1, EPHB2, PAK6, CDC42, PAK7, MAPK1, SEMA4F, RGS3, RAC1, RHOA, SEMA4C, SEMA3A, SEMA4D, ABL1, NFATC3, RASA1, SRGAP2, NFATC1
P00052:TGF-beta signaling pathway	29	6.76E-06	3.07E-04	FOXA2, FOXM1, FOXK2, FOXO3, TGFB1, ATF2, CDC42, FOS, JUND, FOXD1, FOSL1, FOXL2, FOXL1, SMAD7, CREBBP, FOXA1, SMAD4, SMAD3, SKI, SMAD2, FOXP2, MAPK1, EP300, MAPK14, JUN, DCP1A, SMURF2, FOXC1, SMURF1
hsa04510:Focal adhesion	25	1.06E-04	7.39E-04	ERBB2, RAF1, ELK1, ITGB3, PTEN, CTNNB1, PAK6, CDC42, MAPK1, IGF1R, PAK7, CRKL, CCND2, ITGA5, JUN, ITGAV, BCL2, VEGFA, RAC1, COL1A2, RHOA, RAP1A, COL1A1, PIK3R3, AKT3
hsa04330:Notch signaling pathway	11	1.20E-04	7.93E-04	KAT2A, DTX4, CTBP2, EP300, HDAC2, KAT2B, HDAC1, DLL4, CREBBP, RBPJ, LFNG
hsa04310:Wnt signaling pathway	20	2.88E-04	0.0018175	PPP2R1B, TBL1XR1, CTBP2, CREBBP, TP53, SMAD4, SMAD3, SMAD2, TCF7L2, CTNNB1, EP300, CCND2, JUN, RAC1, RHOA, CAMK2D, NFATC3, FOSL1, MYC, NFATC1
P04393:Ras Pathway	19	1.02E-04	0.0030797	MAP2K4, RHOQ, RAF1, ELK1, STAT1, SRF, STAT3, ATF2, PAK6, CDC42, MAPK1, PAK7, STAT4, ETS1, MAPK14, JUN, RAC1, RHOA, AKT3
hsa04010:MAPK signaling pathway	28	6.36E-04	0.0038352	ELK1, NFKB1, NFKB2, SRF, TGFB1, ATF2, CDC42, MAX, FOS, ELK4, RAC1, JUND, FAS, TRAF6, MYC, AKT3, RASA1, RELA, MAP2K4, TP53, RAF1, DUSP4, MAPK1, CRKL, MAPK14, JUN, MAPK8IP3, RAP1A
hsa04660:T cell receptor signaling pathway	15	0.001351973	0.0078049	RELA, RAF1, NFKB1, PAK6, CDC42, FOS, PAK7, MAPK1, MAPK14, JUN, RHOA, PIK3R3, NFATC3, AKT3, NFATC1
hsa04662:B cell receptor signaling pathway	12	0.001608256	0.0089092	MAPK1, FOS, LYN, RELA, JUN, RAC1, RAF1, NFKB1, PIK3R3, NFATC3, AKT3, NFATC1
P00036:Interleukin signaling pathway	31	4.85E-04	0.0109689	IL16, FOXA2, STAT5A, FOXM1, FOXK2, SPI1, ELK1, FOXO3, SRF, STAT6, FOS, STAT4, ELK4, IL10RA, MYC, FOXD1, RASA1, AKT3, STX6, FOXL2, FOXL1, FOXA1, RAF1, IL6R, STAT1, STAT3, STAT2, FOXP2, MAPK1, CDKN1A, FOXC1
hsa04620:Toll-like receptor signaling pathway	14	0.002127613	0.0113221	RELA, MAP2K4, CXCL9, NFKB1, STAT1, FOS, MAPK1, JUN, MAPK14, RAC1, IRF3, TRAF6, PIK3R3, AKT3
P00038:JAK/STAT signaling pathway	8	8.65E-04	0.0130387	STAT6, STAT4, MAPK14, ZMIZ1, STAT5A, STAT1, STAT3, STAT2
hsa04115:p53 signaling pathway	11	0.002548465	0.0130507	CDKN1A, CCND2, BAI1, TP53, MDM2, CDK6, CHEK1, FAS, PTEN, ATM, SESN3
hsa04710:Circadian rhythm	5	0.003595904	0.0171184	NPAS2, PER2, BHLHE40, ARNTL, CLOCK
hsa04062:Chemokine signaling pathway	20	0.00383831	0.0176606	LYN, GNAI1, RELA, CXCL9, RAF1, NFKB1, FOXO3, STAT1, STAT3, CCL26, STAT2, CDC42, MAPK1, CCR7, CRKL, RAC1, RHOA, RAP1A, PIK3R3, AKT3
hsa04150:mTOR signaling pathway	9	0.005102275	0.0226755	EIF4B, MAPK1, EIF4EBP1, HIF1A, TSC1, VEGFA, RICTOR, PIK3R3, AKT3
P00047:PDGF signaling pathway	26	0.00209382	0.0235602	ERG, ELF1, STAT5A, GABPA, RAF1, ELK1, STAT1, SRF, STAT3, USF2, STAT2, STAT6, MAPK1, FOS, STAT4, FLI1, ETS1, ELK4, JUN, RHOA, RAB11A, PIK3R3, MYC, RASA1, AKT3, SRGAP2
hsa04320:Dorso-ventral axis formation	6	0.008453328	0.035126	MAPK1, ETV7, ETS1, ETS2, CPEB1, ETV6
hsa05120:Epithelial cell signaling in Helicobacter pylori infection	10	0.008362331	0.0358193	CDC42, TJP1, ADAM10, LYN, RELA, MAPK14, JUN, MAP2K4, RAC1, NFKB1
P00018:EGF receptor signaling pathway	21	0.004301336	0.0384672	PHLDB1, YWHAZ, ERBB3, STAT5A, ERBB2, MAP2K4, RAF1, RHOQ, STAT1, STAT3, ATM, STAT2, STAT6, CDC42, MAPK1, STAT4, YWHAH, MAPK14, RAC1, AKT3, RASA1
hsa05016:Huntington's disease	18	0.012404681	0.0497502	CREB1, RCOR1, PPARG, CREBBP, TP53, TBP, REST, POLR2A, VDAC1, NRF1, SIN3A, HDAC2, EP300, SP1, HDAC1, GRIN2B, SDHC, NDUFS1
